# RNAblueprint: flexible multiple target nucleic acid sequence design

**DOI:** 10.1093/bioinformatics/btx263

**Published:** 2017-04-25

**Authors:** Stefan Hammer, Birgit Tschiatschek, Christoph Flamm, Ivo L Hofacker, Sven Findeiß

**Affiliations:** 1 Faculty of Chemistry, Department of Theoretical Chemistry, University of Vienna, Vienna, Austria; 2 Faculty of Computer Science, Research Group Bioinformatics and Computational Biology, University of Vienna, Vienna, Austria; 3 Research Network Chemistry Meets Microbiology, University of Vienna, Vienna, Austria; 4 Center for Non-Coding RNA in Technology and Health, University of Copenhagen, Copenhagen, Denmark

## Abstract

**Motivation:**

Realizing the value of synthetic biology in biotechnology and medicine requires the design of molecules with specialized functions. Due to its close structure to function relationship, and the availability of good structure prediction methods and energy models, RNA is perfectly suited to be synthetically engineered with predefined properties. However, currently available RNA design tools cannot be easily adapted to accommodate new design specifications. Furthermore, complicated sampling and optimization methods are often developed to suit a specific RNA design goal, adding to their inflexibility.

**Results:**

We developed a C ++  library implementing a graph coloring approach to stochastically sample sequences compatible with structural and sequence constraints from the typically very large solution space. The approach allows to specify and explore the solution space in a well defined way. Our library also guarantees uniform sampling, which makes optimization runs performant by not only avoiding re-evaluation of already found solutions, but also by raising the probability of finding better solutions for long optimization runs. We show that our software can be combined with any other software package to allow diverse RNA design applications. Scripting interfaces allow the easy adaption of existing code to accommodate new scenarios, making the whole design process very flexible. We implemented example design approaches written in Python to demonstrate these advantages.

**Availability and implementation:**

RNAblueprint, Python implementations and benchmark datasets are available at github: https://github.com/ViennaRNA.

**Supplementary information:**

Supplementary data are available at *Bioinformatics* online.

## 1 Introduction

RNA molecules are omnipresent in all domains of life. They execute diverse functions including small molecule sensing, signal transduction and gene regulation. RNA is a molecule well-suited for designing with predefined functionality. This is mainly due to its close structure to function relationship and the physio-chemically grounded energy models for straightforward *in silico* calculations at the level of secondary structure. In recent years, due to the advent of synthetic biology, more researchers are focusing on the design of synthetic RNAs. There has been increasing success in modifying existing systems and incorporating novel functionality in RNAs within a cellular context (Chappell *et al.*, 2015; Espah-Borujeni *et al.*, 2015; Green *et al.*, 2014; Rodrigo *et al.*, 2012)

To produce an RNA molecule with a prescribed function, the close structure to function relationship must be incorporated into the design process, along with a rationally defined specification of the structure performing that function. In the simplest form one could generate all 4^*n*^ possible nucleic acid sequences of length *n* and test for each sequence if it fulfills the design criteria, e.g. its most stable fold is the structure of interest. Only a small subset of all possible sequences will be actually able to fold at all into the target structure and it is therefore favorable to generate those candidate sequences at least comply with the structural constraints, i.e. are able to fold into the defined structure. Generating only those sequences able and likely to fold into the target structure is known as the ‘inverse folding problem’ (Hofacker *et al.*, 1994) where the applied structural constraints reduce the size of the solution space to be investigated. Biologically active RNA molecules such as aptamers or ribozymes frequently require specific nucleotide patterns in binding or catalytic domains. Therefore, the designed RNA must also comply with certain sequence constraints. Several computational tools capable of solving this hard combinatorial optimization problem have been published. These tools differ mainly in how the initial sequence is selected and which search strategy, e.g. stochastic local or global search, is applied (see Supplementary Table S1). Both algorithmic characteristics have a big impact on the success of the optimization.

A variety of RNA molecules, natural as well as artificial, have been described that exploit structural change as their functional mechanism. Usually, the structural switching of these RNAs between an inactive and the active conformation is induced by an external trigger, which can be as diverse as temperature, small organic molecules, or other small RNAs (Berens and Suess, 2015). The design of such RNA devices requires finding a sequence compatible with two or more structural constraints. Designing a bi-stable RNA was first solved by Flamm *et al.* (2001) using a graph coloring approach. Recent tools can now also design multi-state (three or more) RNA molecules (Höner zu Siederdissen *et al.*, 2013; Lyngso *et al.*, 2012; Taneda, 2015; Wolfe and Pierce, 2015; Zadeh *et al.*, 2011a). The most recent version of the RNAiFold server seems to accept more than two target structures, this extension is however not yet described in the latest publication (Garcia-Martin *et al.*, 2015). Algorithms able to handle multi-state as well as multi-sequence folding and pseudoknotted structures are required if multiple RNA molecules are used as triggers. The latter are implemented in the NUPACK design and analysis framework (Zadeh *et al.*, 2011b).

Sampling sequences compatible with multiple structural constraints can be achieved using a complex graph coloring algorithm (Abfalter *et al.*, 2003; Höner zu Siederdissen *et al.*, 2013). It solves this problem in a defined way where each solution is drawn statistically fairly with equal probability. In contrast, other sampling approaches use ad hoc sampling heuristics that introduce biases and often exhibit undefined runtime complexities (Lyngso *et al.*, 2012; Taneda, 2015). Thus, good solutions may be missed because the sampled part of the solution space is not clearly specified and therefore cannot be fully explored. Furthermore, frequent re-evaluation of already discovered solutions due to biased sampling leads to inefficient optimization, especially if the calculation of the objective involves demanding computations such as pseudoknot structure prediction.

A review of the literature revealed that published RNA designs were either achieved by manual ad hoc approaches or very specific software implementations, which can handle only restricted design problems on a case-by-case basis (Isaacs *et al.*, 2004; Neupert *et al.*, 2008; Qi *et al.*, 2012; Rodrigo and Jaramillo, 2014; Wachsmuth *et al.*, 2013). Very recent publications focus on the flexibility of the design approach and provide methods and interfaces to allow the specification of broader objectives (Höner zu Siederdissen *et al.*, 2013; Taneda, 2015). However, the diversity of the objectives is still limited and introducing a new feature in the objective function requires changes in the program code (some of which are closed source). Furthermore, the mechanisms of optimization in existing tools are always predefined and very rigid.

To address these limitations, we developed RNAblueprint which solves the problem of sampling RNA sequences compatible with multiple structural and sequence constraints in a well defined way. The library is able to specify the runtime complexity and memory requirements of the problem for any given constraints, calculate the number of possible solutions, and to stochastically sample uniformly from all solutions. Furthermore, our technique can be easily integrated into existing tools, henceforth making it possible to focus on the formulation of the objective function as the most crucial part of the design process. Until now this aspect was largely neglected, even though the objective describes best how the design should function. The actual optimization process is performed using the scripting interface, where we offer predefined solutions but also give the user the opportunity to easily implement new ideas without having to change the source code of the core library. Next to the well defined way of describing and exploring the solution space, this flexibility is a major advantage of our approach.

With our framework, in addition to predicting RNA structure and RNA–RNA interactions, and allowing for pseudoknot incorporation (Janssen and Giegerich, 2015; Lorenz *et al.*, 2011; Zadeh *et al.*, 2011a,b) recent methods for the calculation of RNA-ligand interactions can also be incorporated (Lorenz *et al.*, 2016). Using RNAblueprint and its scripting interface we here implemented a multi-state design, which we used to analyze and benchmark our software. The developed software allows us to effectively solve problems including the design of translational and transcriptional on/off switches, triggered by diverse inputs such as small RNAs, ligands, temperature, salt concentration or proteins. RNAblueprint can also be used to specify the design of RNA or DNA scaffolds in synthetic biology, and to construct RNA/DNA origami.

## 2 Approach

An RNA sequence $x=\{x_{1},x_{2},x_{3},\ldots ,x_{n}\}$ is constructed from a set of monomers $x_{i}\in A=\{A,U,G,C\}$ that can interact by forming base pairs (*i*, *j*), 1≤i<j≤n where *i* and *j* are positions separated by at least three bases and $\left(x_{i},x_{j})\in B=\{AU,UA,GC,CG,GU,UG\right\}$ the set of allowed base pairs with B⊂A×A. A set of base pairs of a sequence *x* is called secondary structure Θ.

**Fig. 1.**
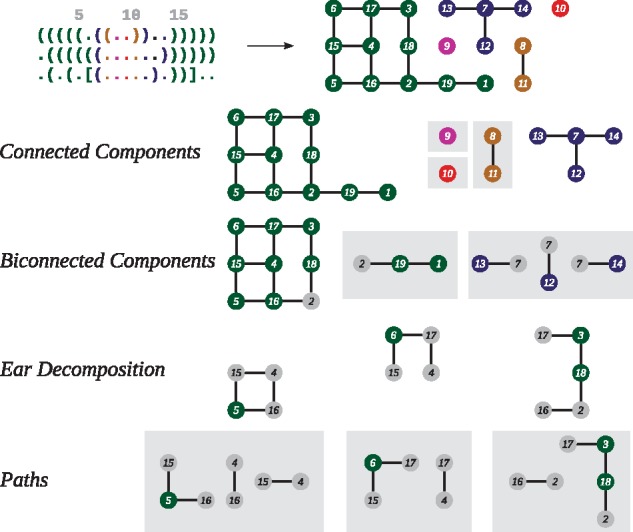
A dependency graph is hierarchically decomposed starting from the top and moving down through four levels to generate a decomposition tree. The dot-bracket strings (top left) denote three structural input constraints which are converted into a dependency graph (top right) by intersecting their circle representations, see Supplementary Figure S1. For an easy visual mapping sequence positions are indicated above the dot-bracket string by an increment of five. Gray boxed subgraphs are not decomposed further as their number of possible colorings can be obtained with the path coloring approach. 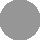 nodes represent articulation points

RNAblueprint implements a method to sample RNA sequences compatible with all structures of a given set $\left\{\Theta_{1},\Theta_{2},\ldots ,\Theta_{M}\right\}$ and sequence constraints $\left\{\Upsilon_{1},\Upsilon_{2},\Upsilon_{3},\ldots ,\Upsilon_{n}\right\}$ where $\Upsilon_{i}\subseteq A$ is the set of allowed nucleotides at position *i*. To be able to uniformly sample from the entire solution space C (which is the set of all *x* compatible with all $\Theta_{t},1\leq t\leq M$, given all sequence constraints $\Upsilon_{i},0\leq i\leq n$), we implemented the graph-theoretical coloring approach developed by Abfalter *et al.* (2003), which is depicted in Figure 1 and described in the following. The goal is to generate sequences that are compatible with a sequence constraint and a set of target structures. Such a design problem is represented as a *dependency graph*G=(V,E) constructed as the union of the circle plot representations of the structural constraints (Supplementary Fig. S1). Each vertex $v_{i}\in V$ of the graph corresponds to a position 1≤i≤n in the sequence to be designed, and the edges *E* represent base pairs (*i*, *j*) that are formed between two vertices. Each base pair occurs at least in one of the input structures. According to the generalized intersection theorem, there exists a solution given the structural constraints, *iff* the resulting graph is bipartite (Flamm *et al.*, 2001). Finally, a coloring or base assignment on a vertex *v_i_* is a single nucleotide $x_{i}\in \Upsilon_{i}$ assigned to the position *i*. Note, that poorly chosen sequence constraints might lead to an unsolvable design problem if they contradict the base pairing pattern enforced by the structural constraints. However, this can already be detected during the graph construction process.

For the design problem with two structural states, Flamm *et al.* (2001) showed that connected components and the underlying sequence positions of the corresponding dependency graph belong to one of the following classes: (i) isolated positions that are unpaired in both structures, (ii) positions that are paired with the same pairing partner in both structures and therefore form paths of length one and (iii) positions that are paired differently in both structures and therefore form paths or cycles. Connected components in (i) and (ii) can be assigned with any element of A and B, respectively. Paths and cycles belonging to (iii) can be stochastically colored by a simple recursion. Furthermore, it was demonstrated how Fibonacci numbers can be used to determine the number of possible solutions for the latter.

Following these results, it is desirable to decompose a more complex dependency graph generated by more than two structural constraints into the aforementioned classes. This decomposition happens at vertices with degree greater than two, denoted as a set of *articulation points*S. Depending on the decomposition algorithm, these nodes are also called cut points or attachment points. As the subsequently explained coloring approach can be very memory and CPU demanding, it is important to follow a specific order on how to decompose the dependency graph into paths. Connected components containing articulation points are decomposed into biconnected components and if they still contain articulation points, they are further decomposed using the ear decomposition algorithm, see Figure 1. An ear decomposition of a graph starting with a path *P*_0_ is a decomposition of its edge set $E=P_{0}\cup \;P_{1}\cup \ldots \cup P_{k}$ where $P_{i+1}$ is a simple path or ear whose endpoints belong to $P_{0}\cup \ldots \cup P_{i}$, but its internal vertices do not (Maon *et al.*, 1986). Our step-wise decomposition approach ensures that the dependency graph falls apart into paths and cycles in a fixed order. As soon as the maximal degree of a subgraph *H* is two, either a path or a circle is reached and further decomposition is terminated. Using this decomposition approach, a binary tree of subgraphs is generated where the complete dependency graph sits at the root and each step of decomposition leads to a fixed order of subgraphs.

**Fig. 2.**
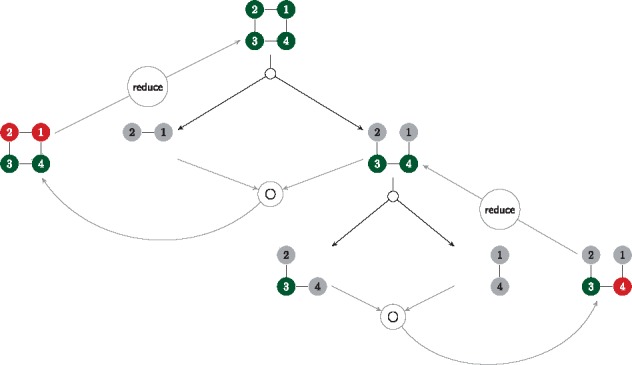
Algorithmic implementation of the decomposition (black arrows) and the reassembly (gray arrows) of a biconnected component. 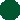 nodes are ordinary nodes and 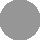 nodes indicate articulation points. 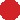 nodes are internalized articulation points which can be converted to ordinary nodes with the reduce function. During the dynamic programming forward recursion, the matrix concatenation operator calculates the number of possible colorings of the combined subgraphs given any assignments on S

After the graph decomposition, the coloring problem therefore reduces to the determination of possible colorings of articulation points (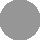 and 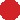 vertices in Fig. 2) in the generated subgraphs *H*. This information can be efficiently calculated by a dynamic programming procedure (Abfalter *et al.*, 2003). Uniform sampling from C can then be achieved by stochastic backtracking. First articulation points are assigned, followed by the sampling of colors for adjacent paths. For ear decompositions this has been described in (Höner zu Siederdissen *et al.*, 2013). In this contribution we describe a generalized approach that covers the dynamic programming for all decomposed components of the dependency graph.

The dynamic programming forward recursion we implemented traverses the binary decomposition tree from the bottom up, ending at the complete assembled dependency graph *G*. For every subgraph *H* the possible colorings for the set of articulation points $S_{H}$ and the according number of available solutions for *H* given these colors are stored in a memorization table during the dynamic programming procedure. The dimension of such a table is determined by $\left|S_{H}\right|$. Since this number differs during the recursive traversal of the graphs in the decomposition tree (smaller graphs are connected at articulation points to larger units) the dimension of the memorization tables also varies. A table dimension itself is indexed by the elements of A. For unbranched paths of length *l* the number of colorings can easily be looked up in the *l*-th power of the pairing matrix P. The memorization table of any other subgraph *H* (parent node in the decomposition tree) is always calculated from the memorization tables of its two smaller constituting graphs (child nodes in the decomposition tree) in a type of concatenation procedure (Fig. 2). The corresponding entries of the articulation points (table dimensions) are first multiplied component-wise and then inserted into the new table. In our implementation the memorization tables are sparse objects and the above construction procedure only increases dimensionality of the tables. The result would be a sparse memorization table with |S| dimensions at the root node of the decomposition tree. To avoid wasting of memory resources, we introduced a dimension reduction step during the successive construction of the memorization tables. This reduction step rests on the observation that whenever the vertex degree of an articulation point in a partially assembled graph is equal to the vertex degree of the corresponding node in the union graph (root node of the decomposition tree) no further subgraph will be ‘attached’ to this particular vertex in subsequent memorization table concatenation operations (see Fig. 2). Hence, the corresponding dimension of the memorization table is collapsed via summing up the values over that internalized articulation point, which shrinks the memorization table and removes the articulation point from the table. This implies that memorization tables for connected components have dimension zero since all articulation points have been internalized and removed via summation. In other words a memorization table with zero dimensions stores the total number of possible colorings for the respective subgraph. The memorization table for the root graph (i.e. the original union graph) therefore stores the size of the solution space, |C|, which is equal to the total number of sequences compatible with the design constraints. With the help of the total number of sequences, the coloring count entries of the memorization tables can be re-interpreted as probabilities, paving the way for uniform sampling approaches.

The sampling procedure works exactly in the opposite order of the memorization table calculation. For each subgraph, articulation points are colored by stochastic backtracking from the probability matrix, which corresponds to the re-interpreted memorization table, followed by the sampling of the graph itself, if it is a path. Otherwise the next hierarchical level of subgraphs is processed. If an articulation point has a base assigned already, this information is used during the stochastic backtracking. Finally, when the last child has been processed, all bases are assigned and a solution was fairly drawn from the complete solution space.

Besides *global sampling*, i.e. generating a completely new sequence all the time, RNAblueprint offers two more procedures to mutate or resample parts of the sequence. *C-local sampling* resets the base assignments of all vertices of a random connected component and draws new colors, i.e. nucleotide assignments, for these vertices. *P-local sampling* randomly selects one path at the leaves of the decomposition tree and resamples only vertices which are not articulation points. This way we ensure the compatibility within a connected component. For both C-local and P-local sampling it can be useful to restrict the random selection of subgraphs by minimal and maximal size constraints or to directly select the connected component or path. The possibility to resample a specific position in the sequence also exists. This either involves a P-local sampling of the path containing the position or, in cases where the selected position corresponds to an articulation point, a C-local sampling of the corresponding connected component. In this way, the ranges of positions to be sampled can be specified. A history of previous sampled sequences is stored, making it convenient to revert to those previous sequences if necessary.

The complexity of our program strongly depends on the number of articulation points |S|. The minimum time complexity O(n) is specified by running the graph decomposition algorithms or path colorings. For every subgraph *H*, the memory and CPU requirements of the dynamic programming coloring approach can be denoted as $O(|A{|}^{\alpha }),\alpha =|S_{H}|$ and $O(|A{|}^{\beta }),\beta =|\underset{h\in C(H)}{\cup }S_{h}|$, respectively. *C*(*H*) represents the set of child subgraphs of *H*. The overall complexity is therefore defined as the sum over all *H*. The latter varies, as the ear decomposition is not done in a deterministic way. It derives from one of many possible spanning trees of the corresponding graph and it follows that *α* and *β* can vary dramatically as investigated in (Höner zu Siederdissen *et al.*, 2013). Therefore, we generate a set of random instances of spanning trees and select the one with lowest *α* and *β* values.

The implementation is written in C++ using the boost graph library and other parts of the boost library available at http://www.boost.org/. Using the SWIG framework, we offer an easy to use Perl and Python scripting interface to the library. Additionally, we developed a Python module so that code can be reused for many central components.

## 3 Materials and methods

### 3.1 Objective function

The original objective function *f*(*x*) proposed by Flamm *et al.* (2001) for two target designs was extended to the multi-target case (Höner zu Siederdissen *et al.*, 2013) and is

(1) $$f(x)=\underset{\text{dominate}\;\text{ensemble}}{\underset{︸}{\sum_{i}^{M}(E(x,\Theta_{i})-G(x))}}+\xi \underset{\text{minimize}\;\text{energy}\;\text{difference}}{\underset{︸}{\sum_{i<j}^{M}(E(x,\Theta_{i})-E(x,\Theta_{j}){)}^{2}}}$$

where *G*(*x*) is the ensemble free energy, $E(x,\Theta_{i})$ is the free energy of the sequence *x* folded into structure Θ_*i*_ and *ξ* is a weighting factor typically set to 1. The first term is to maximize the energy contribution of each target structure to the ensemble to achieve dominance whereas the second term is to minimize the energy difference of target structures to get them to the same energy level. In Taneda (2015) the latter was changed to $\sum_{i<j}^{M}|E(x,\Theta_{i})-E(x,\Theta_{j})|$ which brings most of the target structure energies close to the minimum free energy (MFE) and outliers are possible. In contrast, the original version attempts to minimize the number of outliers and therefore the distance to the MFE of all states might be higher. Either way, weighting of the two terms is essential in single objective approaches. Although objective function (1) showed good performance on two-target designs, the straight-forward extension to three or more structures neglects the varying number of target structures. We therefore modified the objective function to

(2) $$\begin{array}{l} f(x)=\frac{1}{M}\sum_{i}^{M}(E(x,\Theta_{i})-G(x))\\ +\xi \frac{2}{M(M-1)}\sum_{i<j}^{M}|E(x,\Theta_{i})-E(x,\Theta_{j})| \end{array}$$

as we sum up *M* elements in the first term and build $\left(\begin{array}{c} M\\ 2 \end{array}\right)$ differences in the latter. With this new objective function, the ratio between the two terms is independent of the number of structures *M*. To preserve the good performance for the two-target structure case and keep the 1:1 ratio between the two terms in the objective we set *ξ* to 0.5.

### 3.2 Benchmark datasets

The number of target structures is only a rough estimate of the complexity of a given design problem. If the given structural constraints have no conflicting base pairs, the complexity of the connected components are just single vertices or paths of length one. If more overlap between the structural constraints exists, paths get longer, and complex subgraphs such as cycles and blocks occur. Based on a published tri-stable switch (Höner zu Siederdissen *et al.*, 2013), which contains only two cycles and eight paths of length one, we generated more complex examples by adding a fourth and fifth structural constraint, see Supplementary Figure S2A–C. These three example inputs of increasing complexity were used to evaluate the implemented sampling procedures of RNAblueprint. The effect of uniform sampling is tested on an extreme example that contains one large and complex connected component and a base pair as well as an unpaired position. To further reduce the solution space size, two sequence constraints were introduced, see Supplementary Figure S2D.

Comparison with existing approaches was performed on the published datasets containing two-, three- and four-target designs as well as pseudoknotted two-target structure examples (Taneda, 2015). The applied optimization is described in Section 3.3.

### 3.3 Multi-state design

To be able to benchmark against existing design software, we implemented an optimization approach consisting of RNAblueprint for uniform sequence sampling, the value of the objective function (2) to determine the cost of a solution, and an adaptive walk. The latter works as follows: Consecutive sequence candidates are generated by randomly applying one of the three sampling methods, i.e. P-local, C-local or global and calculating the cost. The new sequence is only kept if the cost is lower than the current best solution. Depending on the chosen method, one randomly selected subgraph (P-local and C-local sampling) or all subgraphs (global sampling) are redrawn. The stop condition was set to 1000, being the maximum number of optimization trails with no cost improvement. An optimization run would therefore be stopped earliest after 1000 sampling steps, which gave reasonable results for design problems with increasing complexity, see Figure 4. To compare this approach to existing multi-target design tools we created 100 solutions for each member of the two-, three- and four-target design datasets described in Taneda (2015). Energy calculations for these datasets were made using the scripting bindings of the ViennaRNA package v2.2.5 (Lorenz *et al.*, 2011). As we are not restricted to nested base pairs in the structural input, the pseudoknotted two-target datasets described in Taneda (2015) were also used with stop condition 100. This is set to be much smaller because the runtime dramatically increases when using the Nupack package v3.0.4 (Zadeh *et al.*, 2011b) for pseudoknotted structure prediction. Furthermore, only 30 solutions were generated for each of the latter benchmark tasks.

## 4 Results and discussion

### 4.1 Effect of uniform sampling

**Fig. 3.**
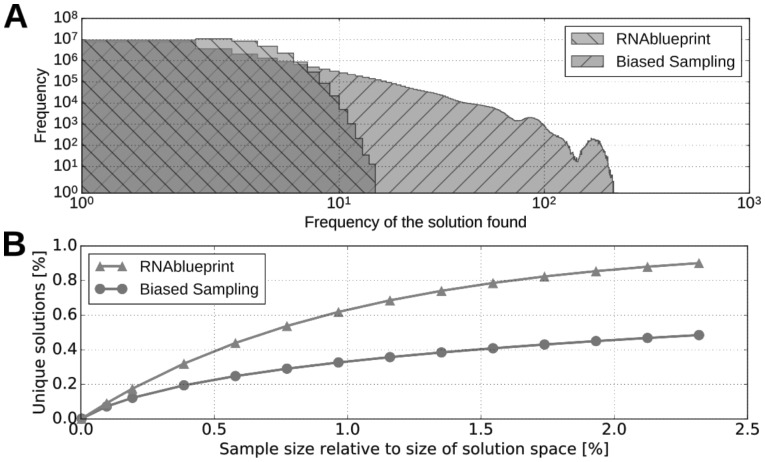
Differences in uniform and biased stochastic sampling shown on a small example with a rather complex dependency graph, see Supplementary Figure S2D. (**A**) The histogram shows how frequent unique sequences were found when sampling completely new candidates using RNAblueprint and the biased sampling method. In total 9.6 × 10^9^ sequences (sample size *d*) were sampled from 4.1 × 10^7^ possible unique sequences (size of solution space |*C*|). While uniform sampling led to an extreme value distribution with the mean (2.57) count being slightly above the relative sample size and the maximum number of times a sequence is rediscovered being 15, biased sampling led to a input specific distorted distribution where a sequence is found 4.78 times on average and 227 times maximal. (**B**) When the sample size was chosen to be much bigger than the solution space (∼ 230%), only about 50% of all possible sequences with biased sampling were obtained for this example, while the uniform sampling method generated about 90%. The performance of RNAblueprint is independent of the underlying problem whereas the curve of the other approaches heavily depends on the properties of the dependency graph

Implementing the complete graph coloring algorithm (Abfalter *et al.*, 2003; Höner zu Siederdissen *et al.*, 2013) and assigning all possible base pairs, RNAblueprint guarantees to uniformly sample the complete solution space. We show that this leads to an extreme value distributed frequency of uniquely found solutions (Fig. 3A). It follows that the solution space, by means of unique solutions generated, can be efficiently explored (Fig. 3B). The expected number of samplings required to explore C is |C| log(|C|) a fact that is known for the related Coupon Collector’s problem (Michael Mitzenmacher, 2005). As the redundancy increases with the sample size *d* (known for the Birthday problem) and |*C*| grows not more than exponentially with *n*, the average number of times sequences are generated when uniform sampling is bounded by log(|C|)∈Θ(n). Thus sequences will typically be sampled at most a linear number of times. The advantage of uniform sampling is most apparent when the amount of generated sequences *d* is large, d≳|C|. In this case, uniform sampling generates a much larger number of unique solutions. To highlight these properties, we implemented a biased sampling method by using the same algorithm as RNAblueprint, but making every backtracking decision uniformly. Thus, we sample all articulation point combinations with the same probability independent of the number of possible solutions of the attached subgraphs. We show that the biased sampling approach produces sequences with varying probabilities heavily dependent on the structure of the dependency graph. Therefore, while generating the same amount of sequences, only a fraction of unique sequences were found compared to RNAblueprint (Fig. 3B). Note, that for very small *d* the curves are almost identical, as expected. However, utilizing an approach that produces many different solutions, avoids heavy re-evaluation of already found sequences.

A simplified version of the graph coloring algorithm was implemented in MODENA (Taneda, 2015). Therein a naïve nucleotide assignment algorithm is used that is able to generate solutions of a design problem but not uniform sampling of the solution space. Furthermore, during the assignment of paired nucleotides without a sequence constraint, the G-U base pair is neglected unless a sequence constraint forces such an assignment. This generates a biased initial population of sequences that are subsequently optimized by applying a genetic algorithm. Unfortunately, MODENA is available as binary only, of which the maximum population size is restricted to 1000 and at least one iteration of the genetic algorithm optimization is enforced. Therefore, we could not compare the effect of the implemented nucleotide assignment algorithm alone. However, their sequence sampling essentially corresponds to our biased sampling method described in the previous paragraph.

The Haskell prototype implementation in Höner zu Siederdissen *et al.* (2013), RNAdesign, used lazy enumeration of all solutions and therefore allows uniform sampling. It opts for O(1) sampling, with low overhead in components. However, this applies only for sufficiently small problems. The way the prototype samples does not scale well for designing sequences with many and complex constraints as each component may get prohibitively large. These limitations get obvious when comparing the memory and CPU requirements of both implementations. While RNAblueprint needs 7 MB and about 0.35 s to generate one compatible sequence (without any optimization) for the complex design example shown in Supplementary Figure S2D the prototype implementation needs 15 GB and about 45.8 s on an Intel i7-6700, 3.4 GHz machine. The memory requirement is not independent of the sample size and further increases during the sampling process. We are aiming on a flexible approach where the sequence sampling step should not be the bottleneck as it might be necessary to generate multiple dependency graphs for exploring sequence and solutions spaces and application of computationally demanding objective functions, e.g. including pseudoknot prediction, will anyway slow down the design process.

In summary, our method is capable of generating sequences with a well defined distribution independent of the input constraints or the sample and solution space size. Note, that RNAblueprint can be easily incorporated in any multi-state design software such as MODENA in order to explore the complete solution space of complex multi-state design problems in an unbiased way.

### 4.2 Sequence sampling

In a typical RNA design scenario, sequences compatible with the structural constraints are scored using an objective function, which gets either minimized or maximized. Thereby, the sequence space is transformed into a landscape of complex and typically unknown structure that needs to be explored. Sampling completely new sequences generates solutions distributed over the complete landscape. This way, for an infinite sampling time the global optimum is always found. However, the optimization is rather slow because in each sampling step the reachable neighborhood is the complete solution space. The solution space of small examples is already of size 4.1 × 10^7^ to 1.4 × 10^14^ (Supplementary Fig. S2) and therefore only a small fraction of all solutions is evaluated during a typical optimization run. The other sampling methods, i.e. P-local and C-local, described in Section 2 dramatically reduced the size of the reachable neighborhood. An adaptive walk using these move steps led to the solution ending up in local minima. The quality of these minima and how fast they were reached depends on the number of nucleotides changed in each step, Supplementary Figure S3.

**Fig. 4.**
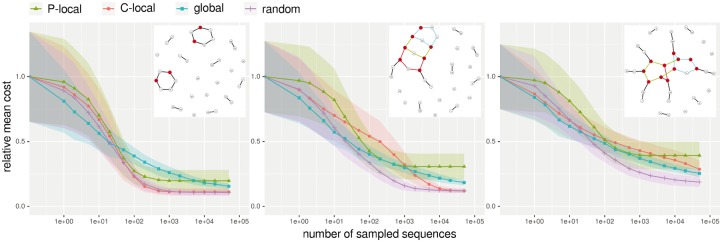
Cost change during the optimization procedure using different move steps and dependency graphs. We minimized function (2) with *ξ* = 0.5 to calculate the cost. The x-axis shows the number of sampled sequences while the y-axis resembles the mean cost from 100 optimization runs, normalized to the mean cost of the initial randomly chosen sequences. For each trend curve the confidence interval (± *σ*) is indicated. Three different move steps (P-local, C-local and global) and an additional run, where one of these moves was randomly picked at every step (random), are compared. At the leftmost plot a very simple dependency graph was generated, only consisting of paths and two cycles, in the middle plot the graph already contains a block and on the right hand side many vertices are captured in one big connected component. The slope of the cost change mainly results from two aspects, the rejection rate and the quality of the newly found solutions. Both are heavily dependent on the size of the move step, therefore we see a change from the left to the right plot, as the move steps of C-local, global and random become bigger, Supplementary Figure S3

In Figure 4, the published three state design example (Höner zu Siederdissen *et al.*, 2013) was extended to four and five input structures. The extension was made in a way that the complexity of the dependency graph from short paths and cycles in the three state example was increased to larger connected components, Supplementary Figure S2. We compared the performance of different sampling methods that differ in the size of their largest move step, see Figure 4. One method, called *global*, always generates a completely new sequence. When sample *C-local* is applied, the assignments of a randomly selected connected component are redrawn. The random selection is weighted by the number of possible solutions associated to the connected components. In contrast, *P-local* resamples only vertices which are not articulation points of a randomly selected path.

If the dependency graph contained only short paths and cycles (three state example), the C-local sampling approach was similar to the P-local sampling, i.e. reached a local minimum relatively fast and the cost converged. The relative mean cost difference between P-local and C-local sampling minima results from the fact that articulation points were redrawn by the latter only. This allowed a maximum step size of up to six nucleotides (complete circle) compared to three nucleotides (longest path), Supplementary Figure S3. The more complex the dependency graph, i.e. the more articulation points and larger connected components exist, the more pronounced this difference between P-local and C-local sampling, Supplementary Figure S3. If one large connected component contained most of the bases (five state example), performing a C-local sampling where all assignments of the large component are most likely reassigned (Supplementary Fig. S3), was similar to a completely new sampled sequence, i.e. the slope of the corresponding curves in Figure 4 are similar. However, the hamming distance to reachable neighbors was different for C-local and global sampling, Supplementary Figure S3. Reaching a local minimum indicates that most likely no further cost improvement can be made using the same sampling method. For the analyzed examples applying a simulated annealing approach, i.e. using an adaptive walk that allows for the acceptance of worse solutions on the way, did only slightly improve the results, see Supplementary Figure S4. Changing the method and thereby changing the move step allows other local minima with better solutions to be reached. Interestingly, our analyses showed that randomly changing the sampling method in each step, *random* in Figure 4, gave significantly better results faster in most cases. We investigated the reachable neighborhood of selected time points during optimization of the four state design example in more detail, Supplementary Figure S5. After 1000 sampling steps, the mean cost of sequences optimized with the random approach was significantly lower than the cost reached with global sampling (student’s *t*-test *P*-value: 10^–^^55^). Furthermore, the number of neighbors with a cost below the current best solution was similar, Supplementary Figure S5. At the end point of the trend curves (after 500 000 sampling trails), C-local and random sampling reached the same mean costs and within their analyzed neighborhood of size 350 600 no better solution was found, Supplementary Figure S5. Interestingly, the sequences optimized with global sampling did not reach the same mean cost and the likelihood of generating a better solution was very low, Supplementary Figure S5. We stress again that these observations are highly dependent on the design problem, e.g. the complexity of the dependency graph and the length of the sequence to be designed. However, we show in the following that applying the random sampling method to a diverse benchmark dataset of nested and pseudoknotted structural input gives reasonably good results.

### 4.3 Impact of normalization and weighting

To analyze the effect of the corrected objective function (2) and the applied optimization procedure we used the recently published benchmark dataset (Taneda, 2015), which consists of two-, three- and four-target design problems as well as three pseudoknotted two-target sets. These examples were either taken from naturally occurring RNAs that are able to switch between structural states or were generated in a way that reachable, sub-optimal structures are taken as input constraints for the design process. RNAblueprint itself does no optimization but rather implements a move set on uniformly sampled sequences. We implemented an adaptive walk that, given a start sequence, randomly selects one of the three sampling methods and applies it to generate the next sequence candidate. The generated sequence is retained if its cost is lower than the best prior solution. On the small examples evaluated in Figure 4, this approach adapted best to the varying complexity of the underlying dependency graphs. To score sequences, we applied an objective function that ensures on one hand that the target structures of a good solution dominate the ensemble while on the other hand the energy difference between the target structures is minimized. In its original version (1), proposed for the two state design case in (Flamm *et al.*, 2001), the corresponding two terms were summed up without any weighting. Designs for two states gave reasonable results compared to other approaches, see Table 1. However, a systematic extension to three or even more states needs individual normalization of the individual terms. Therefore, we proposed a corrected objective function (2), which is adjusted to the good performing original two state objective. Especially for the four structure designs this yielded a significant improvement over the original one, see Table 1. Note, when using a multi-objective approach it is assumed that the weighting is implicitly found during optimization (Taneda, 2015).

**Table 1.** Comparison of currently available approaches to solve multi-target designs

Nested Structure Input													Pseudoknotted Structure Input					
	RNAblueprint						MODENA^a^			Frnakenstein^a^			RNAblueprint			MODENA^a^		
	*original*			*corrected*														
	2str	3str	4str	2str	3str	4str	2str	3str	4str	2str	3str	4str	LE80	PK60	PK80	LE80	PK60	PK80
*μ*(*δe*_1_)	**0.28**	0.22	1.46	0.31	**0.10**	**0.48**	0.38	0.27	0.84	0.35	0.39	0.92	**0.82**	**0.03**	**0.15**	0.89	0.12	0.29
$\tilde{x}(\delta e_{1})$	**0.00**	**0.00**	0.70	**0.00**	**0.00**	**0.05**	0.10	**0.00**	0.39	0.10	0.10	0.55	0.30	**0.00**	**0.00**	**0.20**	**0.00**	**0.00**
*μ*(*δe*_2_)	**0.34**	0.43	1.96	0.36	**0.26**	**1.21**	0.76	0.54	1.78	1.09	0.96	1.89	**1.09**	**0.08**	**0.17**	1.22	0.32	0.56
$\tilde{x}(\delta e_{2})$	**0.00**	0.20	1.30	**0.00**	**0.10**	**0.80**	0.50	0.30	1.40	0.60	0.80	1.60	**0.55**	**0.00**	**0.00**	**0.55**	**0.00**	0.05

* Results of two-, three- and four-target designs are shown. For RNAblueprint and MODENA two-target designs of pseudoknotted structures are also presented. For each resulting sequence, we evaluated the difference between the most stable target structure to the ground state (*δe*_1_) and the highest energy target structure to the ground state (*δe*_2_). The mean (*μ*) and median ($\tilde{x}$) energy difference for 100 and 30 generated sequences is presented for the nested and pseudoknotted structure input, respectively. Performance of the individual sequences is listed in Supplementary Tables S2–S7. Boldface values highlight the best performing approach on a specific dataset. For RNAblueprint the values for the *original* (1) and *corrected* (2) objective functions are listed.

a Values taken from the original publication (Taneda, 2015).

Comparing the results of our naïve optimization procedure with multi-objective approaches that implement complex genetic algorithms to optimize sequences we performed similar or even better on the benchmark dataset as measured by *δe*_1_, i.e. the difference of the lowest energy target structure to the ground state and *δe*_2_, i.e. the difference between the ground state and the highest energy target structure, on the benchmark dataset. Furthermore, we also compared how often the desired target structures are energetically equal to the predicted MFE structure, see Supplementary Tables S2–S7. These values are termed *n_i_*, *i* being the number of target structures with equal energy to the MFE. Given this benchmark measure, MODENA and RNAblueprint performed similarly. A notable difference between our approach and MODENA is that the latter uses a genetic algorithm to optimize a population of 500 individuals of which the best 100 are taken, while we performed 100 independent optimizations. We expect to get similar sequences from a population-based approach while the solutions generated with our approach are extremely diverse.

Although *δe*_1_, *δe*_2_ and *n_i_* together are a good measure of the solution quality of this specific design problem, they do not describe the actual functionality of the switch *in vitro* or *in vivo*. An objective function describing every aspect necessary to create a functional switch might contain many more features, some of which cannot easily be calculated. Furthermore, it is questionable whether the creation of 100 solutions is even useful. It might be better to run the optimization longer and retrieve 10-20 heterogeneous solutions, as this is a more realistic number for experimental validation.

### 4.4 Flexibility matters

Three example objective functions were proposed by Flamm and coworkers to design two-state temperature and structural switches (Flamm *et al.*, 2001). Those objectives have been adapted to multi-state design and are still used to benchmark new software (Höner zu Siederdissen *et al.*, 2013; Taneda, 2015). MODENA enables the user for the first time to choose from a catalog of different structure prediction methods to calculate features of a given sequence and derive new objectives. However, this catalog is fixed and therefore the complete functionality of the applied software might not be available. This is especially true for recent developments, such as the soft constraint framework implemented in the ViennaRNA package (Lorenz *et al.*, 2016) and the test tube ensemble defect available in NUPACK (Wolfe and Pierce, 2015). Furthermore, the methods to optimize sequences, in the case of MODENA by applying a genetic algorithm, cannot be changed. Therefore, we implemented RNAblueprint as a library and equipped this sequence generator with a flexible scripting interface where the user can easily implement its own optimization procedures and come up with new objective functions. Thus, features such as molecule concentrations, specific nucleotide compositions, or various probabilities can be captured in the design process.

## 5 Conclusion

We have developed a software solution that makes it possible to uniformly sample RNA sequences compatible with structural and sequence constraints. Sampling in an uniform way from a well defined solution space ensures to efficiently investigate the entire solution space and avoids heavy re-evaluation of repeatedly generated sequences. Therefore, it is possible to review many more solutions, which potentially leads to better results. We are currently investigating how to adapt the graph coloring algorithm to implement other desired sampling distributions, such as Boltzmann sampling according to a state energy model in a manner similar to what is done for single target design in IncaRNAtion (Reinharz *et al.*, 2013). This way promising sequences that are able and likely to fold into the target structures would be already favored during the sampling procedure.

Scripting interfaces make it easy to freely combine different optimization algorithms and to incorporate evaluations of different software packages into the objective function. We used the NUPACK and the ViennaRNA package to design multi-stable RNA structures with and without pseudoknots, respectively. With the scripting interface, any software such as the recently published RNA shapes studio (Janssen and Giegerich, 2015) and the approach by Wolfe and Pierce to reduce the amount of unwanted complexes when designing interacting molecules (Wolfe and Pierce, 2015), can be easily integrated. As the correct sequence generation problem for a set of fixed structural constraints is now efficiently solved, further research can focus on the challenging task of finding objective functions that better describe the goals and functions of RNA molecules. Using RNAblueprint it is now feasible to explore a much broader range of objectives and it is easy to adapt and recombine existing software and optimization techniques to generate an RNA molecule that perfectly suits the specific needs and goals of the task.

We illustrated the usefulness of our approach with typical but small sample applications. A general solution for solving all the diverse RNA design problems does not exist and there is also no universal way how to benchmark existing tools or novel approaches against each other. Applied measurements heavily depend on the goal and the objective of the design and therefore user knowledge is always necessary to choose an appropriate optimization method, move set and objective function.

## Supplementary Material

Supplementary Data
