# Supplementary material for: RNAblueprint: flexible multiple target nucleic acid sequence design
Source: Bioinformatics. 2017 Apr 25;33(18):2850–8. doi: 10.1093/bioinformatics/btx263 (PMC5870862; doi:10.1093/bioinformatics/btx263)
Supplement: Supplementary Data [file btx263_supplement.pdf]

Supplementary Material
RNAblueprint: Flexible multiple target nucleic acid sequence design

Stefan Hammer, Birgit Tschiatschek, Christoph Flamm, Ivo L. Hofacker and Sven Findeiß

Contents

1 Supplementary Text **3**

1.1 Local neighborhood of various move steps 3

2 Supplementary Figures **5**

3 Supplementary Tables **9**

1 Supplementary Text

1.1 Local neighborhood of various move steps

To get a better understanding of the solution landscape based on the introduced move steps, we analyzed the local neighborhood of three small examples with dependency graphs of varying complexity shown in Supplementary Figure 2. Using one of the introduced sampling methods (*global*, *C-local* and *P-local*, see section 2 in the main text), the local neighborhood was explored by stochastic sampling. The analysis includes the actual hamming distance to the start sequence (Supplementary Figure 3), and the cost change (Supplementary Figure 5) for the two parts of the multi-state objective function (formula (2) in the main text). Additionally the *random* move, where one of the four sampling methods is chosen randomly at each step was investigated. For C-local, 85% of the reachable neighborhood, i.e. 3506 neighbors, was generated for each of the 100 sequences. The same absolute number was used for the global and random approach. With the P-local move, only very few neighbors can be reached, therefore we sampled as many solutions as possible using an exit condition.

The hamming distance describes the size of the move step in terms of actually changed nucleotides, see Supplementary Figure 3. The distribution of these distances for any move step was very much dependent on the structure of the dependency graph. For the four and five structure example the C-local move always showed a flat distribution at smaller hamming distances and one defined peak at a certain distance. This results from the fact that the corresponding dependency graphs consisted of one bigger connected component in addition to several small ones (see Supplementary Figure 2). The connected components could be divided into two disjoint sets due to the bipartite property of the base assignments. If the coloring pattern of the disjoint sets of the bigger component was changed in a way that the coloring switches, all nucleotides of this big connected component are changed, resulting in the defined peak with a hamming distance of exactly the size of this component. If the sets maintained the coloring pattern, we obtained a flat distribution of several smaller distances. Global sampling of the full sequence resulted in similar peaks, however with a shift towards higher distances, as all the smaller connected components are also resampled at every move. The peaks at higher distances show a more even distribution for the same reason. In the analyzed examples, no decomposed path was longer than three nucleotides, excluding special vertices. Therefore, we only obtained hamming distances between 0 and 3 with the P-local approach. Sampling with a randomly picked move step resulted in a very nice superimposition of all the hamming distance distributions, see Supplementary Figure 3.

We further investigated the cost change from a start sequence to its local neighborhood reachable by applying the described sampling methods, Supplementary Figure 5. This is depicted in two-dimensional density plots as cost changes for the two parts of the multi state objective function (objective 1 and objective 2) at the x- and y-axis. The weighted overall cost change can be obtained by following the inclined lines. The purple line indicates neighbors with a constant overall cost, the scale of the actual improvement or decline can be read from the x-axis. From left to right, plots with further optimized sequences obtained from different time steps of Figure 4 were used as start sequences for the analysis. The degree of optimization is therefore measured as “number of sampled sequences”.

In the most left plot (number of sampled sequences = 0), the local neighborhood showed a quite similar distribution in terms of cost improvements on objective 1 and objective 2 for any sampling method. After 100 iterations of optimization, C-local showed the highest number of neighbors with better costs (number in purple box), furthermore the cost improvement possible for individual neighbors was highest for the C-local approach. Both might result from the low quality of the optimized sequences compared to the other approaches. When analyzing even more optimized sequences after 1000 steps, the C-local move still showed highest number of better neighbors. However, the cost improvement possible was quite similar between the various methods. Only with the P-local approach, the cost could not be substantially improved as the local minimum had almost been reached. After $5 \cdot 10^5$ iterations no better solutions

could be obtained for the C-local and P-local approach as the optimization appeared to have reached a local minimum. Overall, P-local sampling behaved similar to C-local sampling, but reached the local minimum of the optimization much earlier due to the smaller size of the reachable neighborhood. In a local minimum no better solution could be obtained with the same move step. Only the global approach could not reach a optimization minimum, as we sampled from the whole solution space with this move. However, better solutions could only rarely be found (see Supplementary Figure 5).

2 Supplementary Figures

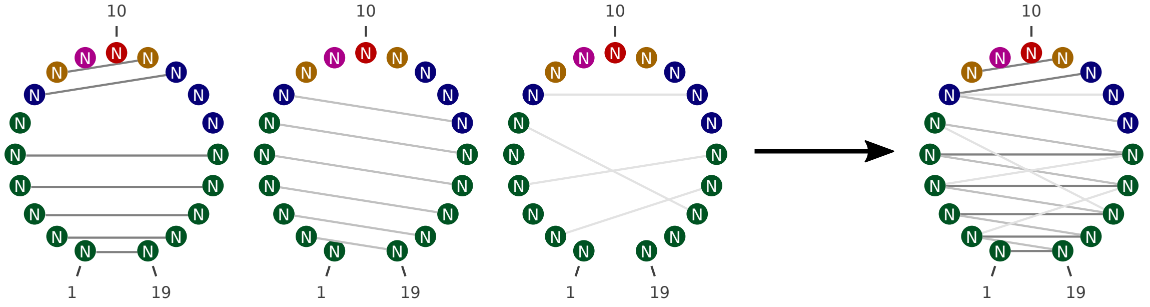

Supplementary Figure 1: The dependency graph G can be generated by the union of the circle plot representations of the given structural constraints. The three input structures in dot-bracket notation are first converted to the circle representation depicted on the left hand side and then the union is formed as shown on the right hand side. Vertices represent bases in a given order along the backbone of the molecule. Edges in different shades of gray represent the base pairs of the three input structures. Colors on the vertices show the different connected components into which the graph can be decomposed. The further decomposition and graph coloring approach of this example is shown in Figure 1 of the main text. Layout by `VARNA`[4]

A)

```
((((....))))....((((....))))........
........((((....((((....))))....))))
((((((((....))))((((....))))....))))

Size of solution space: 1.42658e+14
```

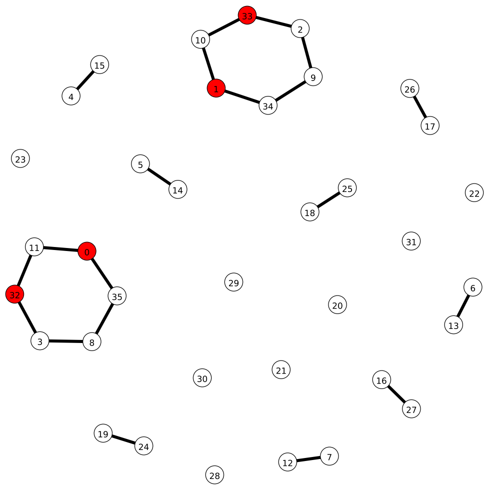

B)

```
((((....))))....((((....))))........
........((((....((((....))))....))))
((((((((....))))((((....))))....))))
.((((....))))...((((....))))........
Size of solution space: 1.24018e+13
```

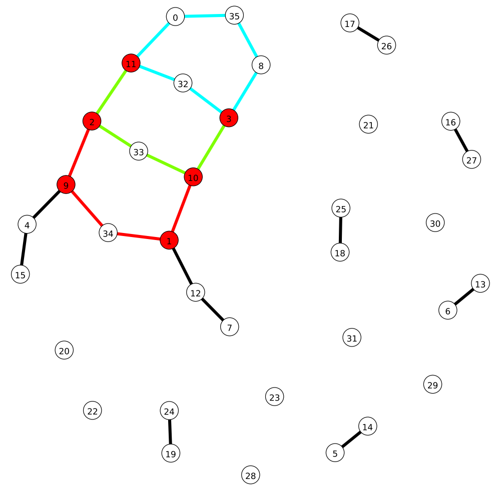

C)

```
((((....))))....((((....))))........
........((((....((((....))))....))))
((((((((....))))((((....))))....))))
.((((....))))...((((....))))........
.((((....))))...((((((........))))))


Size of solution space: 7.08853e+10
```

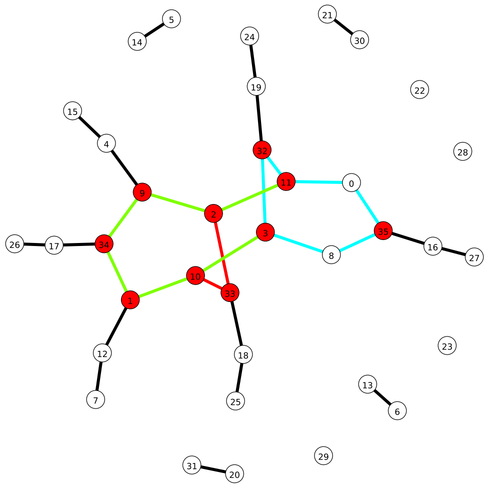

D)

```
.((((((..((....)).....))))))............
..........((.(((....((......))...))).)).
((((((..........(((((......)))))..))))))
.........(.((((((.............)).)))).).
.....((((..(((....))).))))..............
.....(((..............)))...............
ANNNNNNNNNNNNNNNNNNNNNNNCNNNNNNNNNNNNNNN
Size of solution space: 4.14142e+07
```

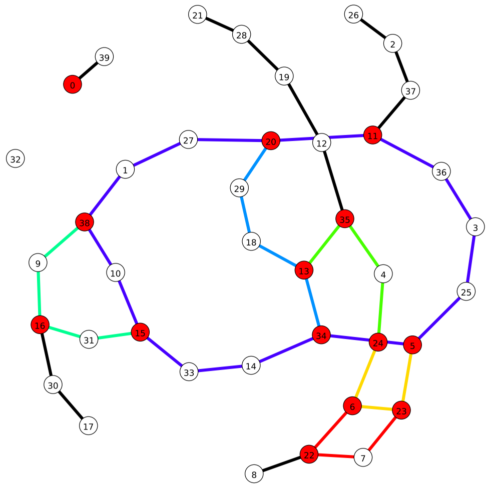

Supplementary Figure 2: Example design inputs and the corresponding graph representations. The depicted examples were used to evaluate different sampling methods provided in `RNAblueprint` (A-C) as well as the fair and unfair sampling (D). The structure and sequence constraint input is shown at the top and the corresponding dependency graph at the bottom in each example. Special vertices are highlighted in red. Edges of circles and paths resulting from the ear decomposition of complex sub-graphs are colored individually.

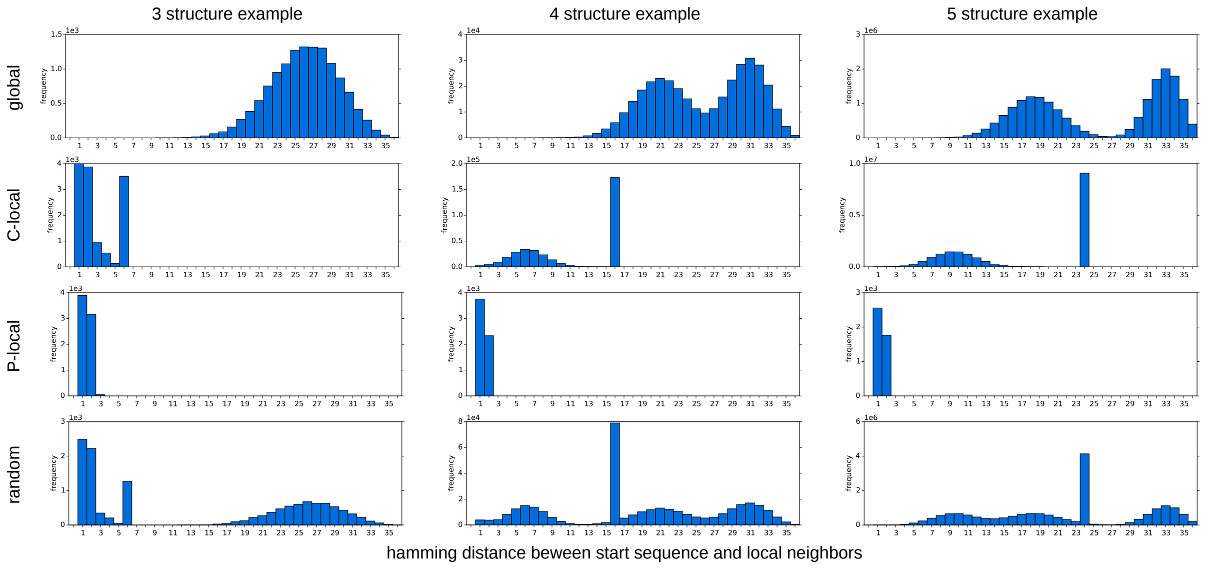

Supplementary Figure 3: Size of different move steps measured as hamming distance between an initial start sequence and most of the reachable neighbors. Rows depict the used sampling method/move steps while columns show the three different small design examples with various complexity (see Supplementary Figure 2A-C). For C-local, 85% of the reachable neighborhood was sampled, while for global and random the same absolute number was used (3str: $1.3 \cdot 10^4$, 4str: $\approx 3.5 \cdot 10^5$, 5str: $\approx 1.6 \cdot 10^7$ sequences). The P-local approach had a much smaller neighborhood, which was sampled with an exit condition to reach most of the neighbors (3str: 7108, 4str: 6076, 5str: $1.3 \cdot 10^4$ sequences).

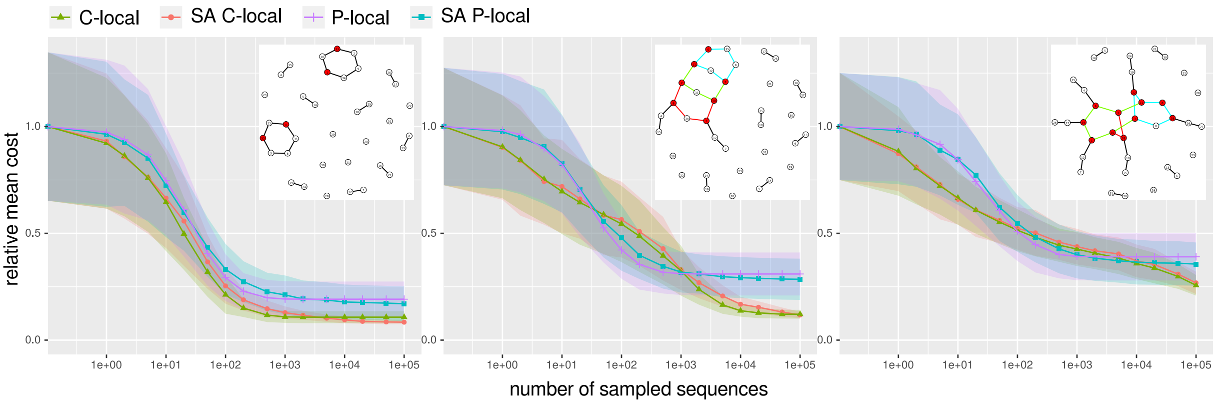

Supplementary Figure 4: Cost change during the optimization procedure using simulated annealing (SA) or an adaptive walk optimization procedure. We minimized function (2) from the main text with $\xi = 0.5$ to calculate the cost. Furthermore, P-local as well as C-local were used as move steps for comparison. The shadowed area depicts the confidence interval ($\pm\sigma$). The x-axis shows the number of sampled sequences while the y-axis resembles the mean cost from 100 optimization runs, normalized to the mean cost of the initial randomly chosen sequences. We used structural inputs with varying complexity listed in Supplementary Figure 2A-C. The simulated annealing approach followed a linear and continuous cooling schedule with $\Delta T = \frac{1}{d}, T_0 = 1$ where d is the number of sampled sequences (x-axis).

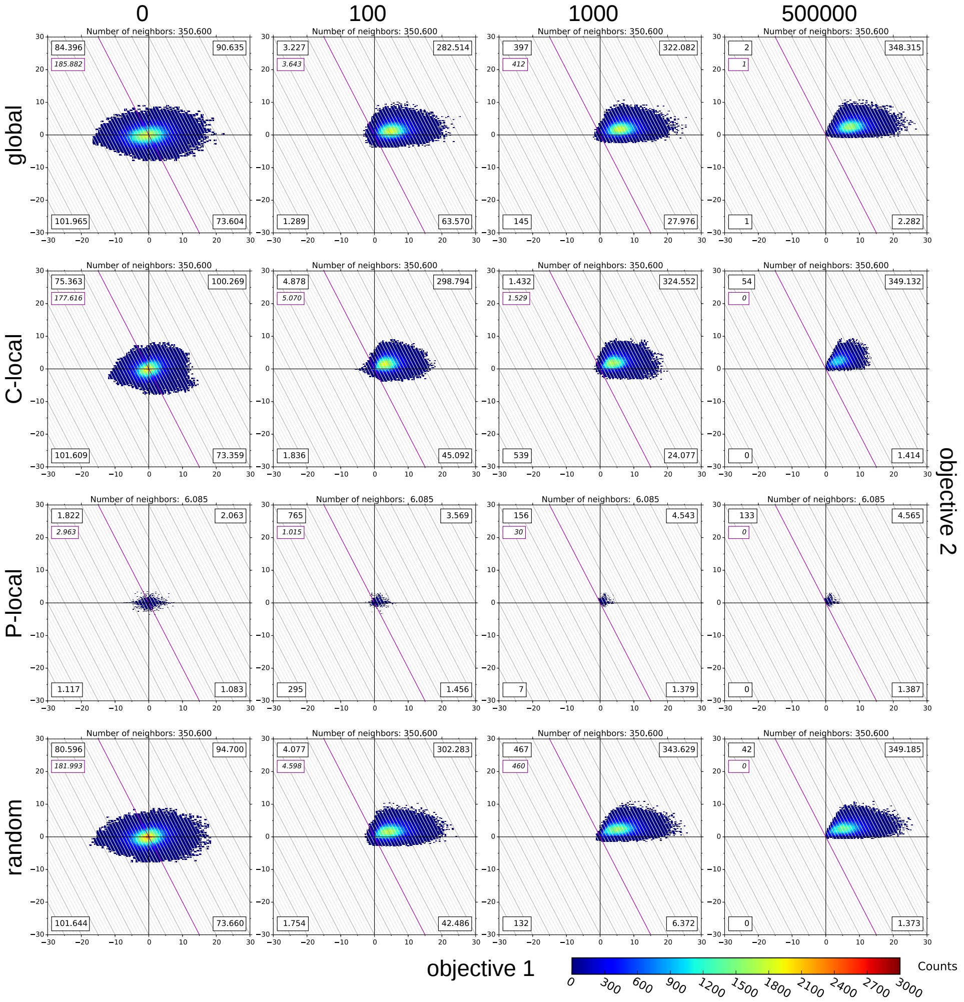

Supplementary Figure 5: Relative cost change to local neighborhood with various move steps on the 4 structure example Supplementary Figure 2B. Each row corresponds to a different sampling method, columns represent the neighborhood of differently optimized sequences obtained from Figure 4 in the main text. The degree of optimization is measured in “number of sampled sequences” during the optimization procedure. The density plots depict the cost change to local neighbors reachable with the used move step. The cost difference is split into changes of the two parts objective 1 and objective 2 on the x-axis and y-axis, respectively. The weighted overall cost change to the start sequence can be obtained by the inclined lines, the purple line indicating unchanged cost. The size of the neighborhood varies, for C-local we sampled 85% of unique neighbors and used the same absolute number for global and random. For the P-local move we sampled as many unique sequences as possible in a reasonable time using an exit condition as this neighborhood is very small. The numbers in the boxes display the count of solutions in this quadrant, the purple box the absolute number of neighbors with a cost change smaller than zero, meaning better solutions than the initial sequence.

3 Supplementary Tables

Supplementary Table 1: Published software to solve the inverse folding problem with single-, two- and multi-target structural input.

Name	Initial Sequence Selection	Search Strategy	Reference
**single-target input**			
RNAinverse	random	stochastic local search	[12]
RNA-SSD	random	stochastic local search	[1]
INFO-RNA	energy optimized	stochastic local search	[3]
RNAexinv	from RNAinverse	stochastic local search	[2]
RNA-ensign	random	global sampling	[17]
IncaRNAtion	seedless	local/global sampling	[20]
DSS-Opt	seedless	Newtonian dynamics simulation and simulated annealing	[19]
EteRNABot	random	stochastic local search	[16]
NUPACK:Design	random	stochastic local search	[5, ?, 25]
ERD	RNA sub-sequences of different structural elements are sampled from natural occurring RNA sequences	evolutionary algorithm	[6]
antaRNA	a graph that represents all possible paths to generate compatible sequences is used	ant colony based optimization	[14, 15]
**two-target input**			
switch.pl	random	stochastic local search	[8]
RiboMaker	random	stochastic local search	[21]
RiboSwitch Calculator	random	genetic algorithm	[7]
RNAiFold2.0	seedless	local or global sampling	[9, 10, 11]
**multi-target input**			
ARDesigner	random	stochastic local search	[22]
Frnakenstein	random or from RNAinverse	genetic algorithm	[18]
MODENA	random	multi objective genetic algorithm	[23, 24]
RNAdesign	random	stochastic local search	[13]

The following tables show the benchmark results summarized in Table 1 in the manuscript. The benchmarks were adapted from Taneda [24] and calculated using the multi-stable design optimization approach and the weighted objective function. For the two-, three- and four-structure inputs (see Methods section in main text) we generated 100 independent solutions using the ViennaRNA with stop condition set to 1000. For the pseudoknotted structure data sets only 30 solutions were generated using the NUPACK package with the stop condition 100.
We used the same measures as in [24] and expanded the table by a probability value. δe_1 and δe_2 are the minimal and maximal energy difference between the evaluated energies of the target structures and the minimum free energy. The values shown in each row of the table are for the solution with the lowest δe_2 and, if multiple solutions existed, also with the the lowest δe_1. Furthermore, $n1, n2, n3, ...nM$ are the number of solutions such that $1, 2, 3, ...M$ of the target structures have the lowest free energy. We also introduced a new measure called "$\sum$ prob", which is the sum of the probabilities of all target structures in the Boltzmann ensemble for the solution picked using the δe_1 and δe_2 values. We furthermore list the time it took to construct the dynamic programming tables and to sample and optimize the sequences in seconds (calculated on VSC3: Intel Xeon E5-2650v2, 2.6 GHz, Ivy Bridge-EP family). Note, the latter includes the energy calculations by ViennaRNA or NUPACK. The column named "max dim" shows the maximal number of dimensions of all dynamic programming tables. This number is the main measure for the complexity and memory requirements or the specific problem (see main text for calculation of the complexity).

Supplementary Table 2: Detailed results of [24] two-target design inputs (SV11 & RNAtabupath dataset)

RNA	l	n1	n2	d1	d2	μ d1	$\tilde{x}$ d1	μ d2	$\tilde{x}$ d2	μ nom	$\tilde{x}$ nom	$\sum$ prob	μ constructing	μ sampling	max dim
alpha operon	130	74	5	0.00	0.00	0.10	0.00	0.28	0.20	7521.18	7071.50	0.46	0.00	185.23	0
amv	145	3	0	0.30	0.40	0.96	0.85	1.16	1.10	9776.94	9353.50	0.22	0.00	308.39	0
attenuator	73	82	71	0.00	0.00	0.08	0.00	0.09	0.00	5149.67	4884.00	0.30	0.00	39.78	0
dsrA	85	2	0	0.10	0.10	1.64	1.50	1.67	1.50	7312.21	6543.00	0.21	0.00	78.00	0
hdv	153	62	44	0.00	0.00	0.19	0.00	0.24	0.10	7583.76	7228.00	0.05	0.00	276.75	0
hiv	280	12	7	0.00	0.00	1.28	1.10	1.36	1.20	15442.83	14653.50	0.02	0.00	2176.04	0
ms2	73	45	35	0.00	0.00	0.33	0.10	0.37	0.20	6092.20	5601.50	0.14	0.00	52.11	0
rb1	148	79	64	0.00	0.00	0.12	0.00	0.16	0.00	7579.62	7279.00	0.10	0.01	230.27	0
rb2	113	42	30	0.00	0.00	0.45	0.20	0.49	0.30	6818.82	6481.50	0.08	0.00	119.27	0
rb3	141	69	42	0.00	0.00	0.14	0.00	0.18	0.10	8740.21	8104.50	0.08	0.01	258.44	0
rb4	146	0	0	2.00	2.00	4.90	4.95	4.99	5.00	9327.93	8506.00	0.00	0.00	319.34	0
rb5	201	73	56	0.00	0.00	0.16	0.00	0.21	0.00	9001.18	8677.00	0.12	0.00	455.17	0
ribD	304	0	0	1.10	1.10	2.99	2.85	3.06	2.95	13307.82	13106.50	0.01	0.00	2078.01	0
s15	74	54	40	0.00	0.00	0.33	0.00	0.37	0.10	5043.62	4666.00	0.25	0.00	40.58	0
sbox	247	0	0	0.70	1.00	1.65	1.20	1.69	1.25	12247.86	11819.50	0.25	0.00	1141.45	0
spliced	56	3	0	0.00	0.20	0.98	1.00	1.05	1.05	4914.67	4528.50	0.19	0.00	31.28	0
sv11	115	9	5	0.00	0.00	1.12	0.90	1.16	1.05	5644.82	5254.50	0.02	0.00	129.32	2
μ		36	23	0.25	0.28							0.15			
$\tilde{x}$		42	7	0.00	0.00							0.12			

Supplementary Table 3: Detailed results of the three-target design inputs (RNAdesign dataset [3str]).

RNA	l	n1	n2	n3	d1	d2	μ d1	$\tilde{x}$ d1	μ d2	$\tilde{x}$ d2	μ nom	$\tilde{x}$ nom	$\sum$ prob	μ constructing	μ sampling	max dim
sq1	100	98	7	0	0.00	0.10	0.00	0.00	0.47	0.30	5975.53	5582.00	0.38	0.00	89.11	0
sq2	100	0	0	0	1.10	1.30	3.51	3.25	4.19	4.20	6957.17	6847.50	0.01	0.00	121.06	1
sq3	100	17	2	0	0.00	0.10	0.60	0.60	1.04	1.00	6980.33	6462.50	0.11	0.00	91.63	2
sq4	100	83	22	5	0.00	0.00	0.08	0.00	0.42	0.30	6566.18	5761.00	0.31	0.00	97.05	2
sq5	100	98	40	13	0.00	0.00	0.00	0.00	0.30	0.20	6353.41	5893.50	0.69	0.00	101.30	0
sq6	100	38	7	0	0.00	0.10	0.40	0.20	1.57	1.30	6569.40	6253.50	0.16	0.00	92.84	0
sq7	100	24	6	1	0.00	0.00	0.64	0.55	1.02	0.90	7195.74	6381.50	0.26	0.00	100.42	3
sq8	100	71	13	3	0.00	0.00	0.19	0.00	0.63	0.50	7156.62	6581.00	0.22	0.00	109.25	0
sq10	100	21	3	0	0.00	0.10	0.68	0.60	1.09	1.10	6510.08	5987.50	0.29	0.00	101.15	1
sq11	100	61	5	2	0.00	0.00	0.21	0.00	0.69	0.60	6276.30	5800.50	0.33	0.00	95.72	0
sq12	100	96	10	0	0.00	0.10	0.01	0.00	0.45	0.30	6201.07	5806.00	0.23	0.00	92.06	0
sq13	100	3	0	0	0.00	1.00	2.78	2.65	3.79	3.60	8529.50	7977.00	0.06	0.01	151.32	4
sq14	100	30	0	0	0.00	0.20	0.69	0.45	1.62	1.50	5814.88	5631.50	0.08	0.00	87.77	0
sq15	100	1	0	0	1.10	1.50	3.04	2.85	4.14	4.00	7987.25	7928.00	0.01	0.00	124.29	3
sq16	100	60	20	7	0.00	0.00	0.18	0.00	0.43	0.30	6868.02	6379.50	0.32	0.00	92.37	0

sq17	100	19	0	0	0.20	0.70	0.74	0.70	1.67	1.50	6226.68	5645.00	0.05	0.00	97.91	0
sq18	100	49	3	0	0.00	0.10	0.58	0.10	1.49	1.40	6430.86	6154.50	0.12	0.00	90.79	0
sq19	100	5	0	0	0.00	0.10	0.80	0.70	1.18	1.10	5899.59	5739.50	0.21	0.00	91.30	2
sq20	100	61	21	4	0.00	0.00	0.24	0.00	0.55	0.40	6217.49	6122.50	0.15	0.01	96.93	0
sq21	100	30	2	0	0.00	0.10	0.69	0.40	1.65	1.50	6281.56	5960.50	0.07	0.00	100.94	0
sq22	100	13	3	0	0.00	0.20	0.96	1.00	1.35	1.30	10181.89	9857.00	0.13	0.00	161.28	2
sq23	100	0	0	0	2.10	2.20	4.42	4.40	5.68	5.60	9113.57	8313.00	0.00	0.00	154.49	3
sq24	100	43	11	3	0.00	0.00	0.40	0.25	0.75	0.70	6759.85	6487.00	0.18	0.00	99.73	1
sq25	100	100	48	19	0.00	0.00	0.00	0.00	0.19	0.15	6919.91	6745.50	0.69	0.00	109.11	0
sq26	100	6	1	0	0.00	0.30	0.87	0.90	1.52	1.50	5995.44	5181.00	0.10	0.00	91.57	0
sq27	100	1	0	0	0.00	0.50	1.24	1.20	1.68	1.60	8398.12	8038.00	0.09	0.00	129.17	2
sq28	100	100	2	0	0.00	0.30	0.00	0.00	0.87	0.80	8230.88	7525.50	0.42	0.00	118.18	0
sq29	100	23	6	2	0.00	0.00	0.62	0.55	1.00	0.90	5672.03	5407.50	0.18	0.00	90.37	1
sq30	100	34	7	2	0.00	0.00	0.54	0.30	0.99	0.85	6888.60	6825.00	0.08	0.00	108.52	2
sq31	100	17	5	1	0.00	0.00	0.59	0.40	1.11	1.00	5486.78	5328.50	0.05	0.00	81.05	0
sq32	100	57	13	3	0.00	0.00	0.38	0.00	0.83	0.70	5432.50	5263.50	0.05	0.00	79.82	0
sq33	100	93	35	18	0.00	0.00	0.02	0.00	0.21	0.20	7759.08	7266.00	0.42	0.00	122.29	0
sq34	100	0	0	0	2.20	2.60	4.99	5.10	5.99	5.90	7943.20	7670.00	0.00	0.00	135.51	2
sq35	100	31	7	2	0.00	0.00	0.59	0.50	1.03	0.85	6722.60	6324.00	0.07	0.00	97.98	0
sq36	100	91	2	0	0.00	0.20	0.03	0.00	0.57	0.40	7397.27	6793.00	0.31	0.00	99.75	0
sq37	100	62	10	1	0.00	0.00	0.18	0.00	0.58	0.50	6480.41	6218.00	0.53	0.01	99.90	0
sq38	100	96	10	0	0.00	0.10	0.01	0.00	0.72	0.70	6178.97	5870.50	0.39	0.00	80.82	2
sq39	100	15	3	0	0.10	0.10	1.33	1.25	2.17	2.05	8087.73	7853.50	0.07	0.00	130.01	2
sq40	100	85	1	0	0.00	1.60	0.05	0.00	1.74	1.70	5812.31	5644.50	0.09	0.00	79.74	0
sq41	100	33	9	1	0.00	0.00	0.47	0.30	0.86	0.70	6434.00	5555.00	0.16	0.00	100.89	0
sq42	100	15	0	0	0.60	1.00	0.96	0.90	2.02	1.70	7347.52	6932.50	0.09	0.00	96.47	0
sq43	100	78	5	0	0.00	0.20	0.10	0.00	1.15	1.10	6068.68	5794.00	0.35	0.00	87.94	0
sq44	100	5	0	0	0.00	0.10	1.19	1.05	1.75	1.70	6358.53	5973.50	0.08	0.00	92.26	1
sq45	100	66	17	5	0.00	0.00	0.14	0.00	0.46	0.40	6132.27	5821.50	0.34	0.00	84.31	0
sq46	100	5	0	0	0.10	0.30	0.94	0.90	1.23	1.30	6289.39	5981.00	0.09	0.00	93.21	1
sq47	100	97	4	0	0.00	0.70	0.01	0.00	1.60	1.10	7376.39	6475.50	0.49	0.00	101.43	0
sq48	100	41	3	0	0.00	0.20	0.37	0.20	1.43	1.30	7333.19	6815.50	0.14	0.01	105.53	2
sq49	100	37	0	0	0.00	0.20	0.32	0.20	1.02	0.90	6034.74	5457.00	0.27	0.00	86.76	0
sq50	100	48	2	0	0.00	0.20	0.22	0.10	0.94	0.80	7113.65	6233.50	0.42	0.00	98.32	0
sq51	100	0	0	0	0.60	1.10	2.43	2.25	3.41	3.25	9160.54	8825.00	0.00	0.01	150.62	3
sq52	100	2	1	0	0.10	0.60	2.43	2.15	3.26	3.25	8423.05	7965.00	0.01	0.00	152.05	2
sq53	100	98	23	13	0.00	0.00	0.00	0.00	0.20	0.20	7125.22	6411.50	0.29	0.00	104.47	0
sq54	100	6	2	1	0.00	0.00	1.44	1.45	1.99	2.00	9938.95	9303.50	0.05	0.00	147.97	2
sq55	100	1	0	0	0.30	0.60	0.96	0.90	1.48	1.40	6368.29	5597.00	0.02	0.01	98.01	0
sq56	100	7	1	0	0.00	0.20	1.24	1.20	1.54	1.50	7552.24	6787.00	0.17	0.00	113.88	0
sq57	100	99	2	0	0.00	0.20	0.00	0.00	0.49	0.40	7489.52	7270.00	0.77	0.00	109.02	0
sq58	100	60	6	0	0.00	0.10	0.23	0.00	1.01	0.90	7231.36	6586.50	0.37	0.00	109.54	0
sq59	100	14	4	3	0.00	0.00	0.89	0.85	1.25	1.30	7664.53	7550.50	0.23	0.01	109.84	3
sq60	100	16	0	0	0.10	0.20	1.10	0.90	1.57	1.40	6759.46	6397.00	0.09	0.00	94.99	0
sq61	100	5	0	0	0.10	0.20	0.47	0.30	0.95	0.70	6632.91	6339.50	0.28	0.00	99.29	0
sq62	100	0	0	0	1.10	1.60	3.21	3.20	4.22	4.15	9448.37	8549.50	0.01	0.01	156.53	4
sq63	100	98	19	14	0.00	0.00	0.00	0.00	0.28	0.30	6861.21	5966.00	0.51	0.00	89.88	0

sq64	100	30	6	0	0.00	0.10	0.73	0.50	1.23	1.10	6767.57	6568.00	0.09	0.01	99.83	1
sq65	100	0	0	0	1.50	1.70	3.10	3.00	4.31	4.10	7224.15	6862.50	0.00	0.00	133.55	3
sq66	100	29	1	0	0.00	0.10	0.70	0.40	1.56	1.50	6621.04	6369.50	0.13	0.01	112.09	1
sq67	100	0	0	0	1.40	3.10	7.66	7.75	9.11	9.15	6991.76	6707.50	0.00	0.01	131.77	5
sq68	100	8	0	0	0.10	0.20	1.29	1.20	1.87	1.80	5628.45	5342.00	0.07	0.01	94.53	2
sq69	100	0	0	0	0.90	1.30	3.47	3.25	4.98	4.90	7484.97	7254.50	0.02	0.00	128.51	2
sq70	100	95	7	0	0.00	0.40	0.03	0.00	0.94	0.60	6528.09	5973.50	0.59	0.00	90.05	0
sq71	100	31	0	0	0.90	1.10	0.53	0.40	2.71	2.75	7094.99	6478.00	0.02	0.00	113.87	0
sq72	100	99	38	24	0.00	0.00	0.00	0.00	0.20	0.20	6428.78	5759.00	0.22	0.00	90.74	0
sq73	100	95	18	1	0.00	0.00	0.01	0.00	0.44	0.40	6848.43	6540.50	0.49	0.01	106.04	2
sq74	100	8	0	0	0.00	0.50	1.33	1.30	2.05	2.00	6568.45	6118.50	0.09	0.00	102.99	1
sq75	100	15	2	0	0.00	0.10	1.03	0.95	1.45	1.30	6588.74	6486.00	0.04	0.00	108.73	2
sq76	100	9	2	0	0.20	0.40	1.73	1.30	2.54	2.30	7323.90	6833.00	0.07	0.00	122.64	1
sq77	100	12	1	0	0.00	0.10	1.25	1.20	1.85	1.70	5951.63	5626.50	0.05	0.00	89.68	0
sq78	100	41	2	0	0.00	0.90	0.47	0.20	2.28	2.10	6518.50	6233.50	0.21	0.01	106.74	1
sq79	100	2	0	0	0.10	0.50	1.88	1.90	2.63	2.70	8657.86	7776.00	0.04	0.01	158.22	3
sq80	100	100	7	2	0.00	0.00	0.00	0.00	0.39	0.40	6933.06	6410.00	0.58	0.00	102.45	0
sq81	100	85	16	7	0.00	0.00	0.03	0.00	0.42	0.30	6684.96	6007.50	0.21	0.00	98.78	0
sq82	100	99	29	18	0.00	0.00	0.00	0.00	0.27	0.20	6867.12	5824.50	0.44	0.00	96.96	0
sq83	100	56	14	2	0.00	0.00	0.25	0.00	0.74	0.60	7350.49	6866.00	0.27	0.00	96.74	0
sq84	100	18	2	1	0.00	0.00	0.41	0.30	0.71	0.70	6109.86	5690.00	0.20	0.00	97.84	0
sq85	100	85	22	13	0.00	0.00	0.04	0.00	0.21	0.20	7482.15	6858.00	0.24	0.00	111.06	0
sq86	100	28	2	0	0.00	0.10	0.60	0.45	1.03	1.00	6010.22	5885.00	0.12	0.00	86.18	0
sq87	100	22	4	0	0.00	0.40	0.47	0.45	1.78	1.60	7865.87	7464.50	0.30	0.00	118.78	0
sq88	100	88	11	1	0.00	0.00	0.03	0.00	0.46	0.40	7470.32	6667.00	0.73	0.00	118.53	0
sq89	100	97	37	21	0.00	0.00	0.01	0.00	0.25	0.20	6052.79	5557.00	0.36	0.00	91.79	0
sq90	100	97	29	6	0.00	0.00	0.02	0.00	0.38	0.30	6660.64	6167.50	0.48	0.00	87.25	0
sq91	100	78	25	9	0.00	0.00	0.09	0.00	0.38	0.30	6520.25	6181.00	0.15	0.00	109.23	1
sq92	100	9	1	0	0.10	0.20	0.68	0.70	1.21	1.30	6748.22	6620.00	0.12	0.00	98.68	0
sq93	100	64	15	7	0.00	0.00	0.15	0.00	0.46	0.30	6222.48	5679.00	0.14	0.00	97.16	0
sq94	100	6	0	0	0.00	0.20	1.07	0.80	1.42	1.30	6180.87	5925.50	0.25	0.00	91.79	0
sq95	100	0	0	0	2.00	2.20	4.69	4.40	5.83	5.70	6537.24	6214.00	0.00	0.04	109.39	6
sq96	100	21	5	0	0.00	0.10	0.49	0.50	0.95	0.90	7243.22	6611.00	0.29	0.00	93.34	0
sq97	100	41	12	1	0.00	0.00	0.52	0.20	1.03	0.95	5482.89	5306.50	0.47	0.01	79.39	0
sq98	100	95	2	1	0.00	0.00	0.01	0.00	0.65	0.50	7096.59	6784.00	0.37	0.00	96.66	2
sq99	100	28	4	2	0.00	0.00	0.45	0.45	0.70	0.70	6321.52	5836.00	0.20	0.00	84.55	0
sq100	100	12	0	0	0.00	0.30	1.02	1.10	1.63	1.50	8625.88	8190.50	0.32	0.00	142.78	2
μ		42	7	2	0.17	0.36							0.22			
$\tilde{x}$		30	3	0	0.00	0.10							0.17			

Supplementary Table 4: Detailed results of the four-target design inputs (RNAdesign dataset [4str]).

RNA	l	n1	n2	n3	n4	d1	d2	μ d1	$\tilde{x}$ d1	μ d2	$\tilde{x}$ d2	μ nom	$\tilde{x}$ nom	$\sum$ prob	μ constructing	μ sampling	max dim

sq1	100	98	11	0	0	0.00	0.30	0.00	0.00	1.15	1.05	7065.64	6290.50	0.27	0.00	107.09	0
sq3	100	0	0	0	0	1.20	3.70	5.11	5.00	7.80	7.80	10313.01	10232.50	0.00	0.01	256.82	4
sq4	100	61	6	1	1	0.00	0.00	0.21	0.00	1.07	0.90	6232.92	5801.00	0.30	0.00	148.02	2
sq5	100	87	12	0	0	0.00	0.20	0.04	0.00	0.75	0.70	6139.30	5751.00	0.49	0.00	158.41	0
sq6	100	27	1	0	0	0.10	0.30	0.61	0.50	1.56	1.45	7384.96	7053.00	0.02	0.00	177.77	0
sq7	100	46	10	2	0	0.00	0.10	0.49	0.10	1.13	0.85	7489.11	6977.50	0.21	0.01	166.58	3
sq8	100	4	0	0	0	0.20	0.70	2.05	1.85	3.49	3.30	7959.74	7439.00	0.02	0.00	206.02	2
sq10	100	10	0	0	0	0.00	0.50	1.05	0.90	2.49	2.30	9071.55	8659.00	0.16	0.00	147.87	2
sq11	100	16	3	1	0	0.00	0.40	0.96	0.90	1.83	1.80	6080.15	5837.50	0.10	0.00	101.82	1
sq12	100	90	0	0	0	0.20	0.60	0.03	0.00	1.62	1.80	6572.47	5914.00	0.09	0.00	107.29	0
sq13	100	1	0	0	0	3.30	4.60	5.87	5.85	8.78	8.65	7085.27	6552.50	0.00	3.17	140.17	10
sq14	100	0	0	0	0	1.20	4.30	3.84	3.70	8.60	8.50	8844.75	8589.50	0.00	0.00	156.09	3
sq15	100	0	0	0	0	4.80	5.80	6.59	6.30	9.05	9.00	9336.95	8877.00	0.00	0.01	169.58	6
sq16	100	91	15	1	1	0.00	0.00	0.04	0.00	0.66	0.70	7928.64	7092.50	0.23	0.00	110.73	0
sq17	100	4	0	0	0	0.40	1.70	2.36	2.10	4.77	4.75	10917.99	10392.50	0.00	0.00	182.14	3
sq18	100	16	1	1	0	0.90	1.10	1.38	1.30	3.40	3.35	6310.94	6095.00	0.01	0.00	104.57	1
sq19	100	1	0	0	0	2.30	3.10	3.64	3.50	7.12	7.00	10726.86	10257.00	0.00	0.01	198.35	5
sq20	100	9	0	0	0	0.20	0.80	1.61	1.35	3.03	2.80	9639.32	9307.50	0.08	0.00	164.04	3
sq21	100	0	0	0	0	4.50	6.30	7.45	7.40	10.40	10.50	6735.71	6625.00	0.00	0.01	124.69	3
sq22	100	5	0	0	0	0.20	1.30	1.51	1.40	3.22	3.00	8779.18	8195.50	0.01	0.00	145.07	2
sq23	100	0	0	0	0	2.20	3.10	3.84	3.85	5.63	5.45	9278.87	8664.50	0.00	0.00	159.84	3
sq24	100	34	5	2	0	0.00	0.10	0.47	0.30	1.28	1.25	8271.60	8009.00	0.26	0.00	132.48	2
sq25	100	84	18	5	1	0.00	0.00	0.06	0.00	0.86	0.80	6895.81	6451.50	0.42	0.01	117.67	0
sq26	100	29	1	0	0	0.10	0.50	0.35	0.30	1.92	1.80	5791.33	5159.00	0.04	0.00	93.58	0
sq27	100	11	0	0	0	0.00	0.10	1.05	1.00	1.90	1.75	9275.04	8329.50	0.12	0.00	168.69	2
sq28	100	74	0	0	0	0.00	0.90	0.14	0.00	3.08	3.30	6816.69	5996.00	0.11	0.00	110.12	0
sq29	100	58	0	0	0	0.00	0.30	0.24	0.00	2.41	2.50	6148.43	5642.00	0.38	0.00	96.53	1
sq30	100	31	0	0	0	0.00	0.90	0.71	0.50	2.96	2.90	8032.09	7731.00	0.06	0.00	164.35	3
sq31	100	2	0	0	0	0.10	1.20	1.53	1.35	3.23	3.15	7308.63	6979.00	0.01	0.00	140.47	2
sq32	100	48	7	3	0	0.00	0.10	0.37	0.15	1.50	1.30	5720.23	5626.50	0.11	0.00	92.73	0
sq33	100	51	2	0	0	0.00	0.40	0.30	0.00	1.45	1.30	7065.44	6734.00	0.09	0.00	143.89	3
sq34	100	0	0	0	0	3.00	3.70	5.83	5.65	7.70	7.80	8253.23	7950.00	0.00	0.00	198.48	3
sq35	100	9	0	0	0	0.00	0.60	1.30	1.10	3.67	3.55	8194.17	7667.50	0.05	0.00	169.42	1
sq36	100	95	2	0	0	0.00	0.30	0.01	0.00	1.41	1.35	7365.93	6961.00	0.20	0.00	137.03	0
sq37	100	0	0	0	0	2.40	3.60	6.38	6.55	9.58	9.70	6678.52	6130.00	0.00	0.01	168.64	5
sq38	100	58	2	0	0	0.00	1.90	0.16	0.00	2.84	2.60	6538.08	6166.50	0.10	0.00	133.12	2
sq39	100	6	0	0	0	0.20	0.70	1.54	1.20	3.11	3.00	8281.63	7869.50	0.06	0.00	212.28	2
sq40	100	63	15	0	0	0.00	1.60	0.19	0.00	2.11	2.00	5824.55	5322.50	0.21	0.00	127.14	0
sq41	100	37	0	0	0	0.00	0.90	0.54	0.40	3.39	3.25	7220.99	6520.00	0.06	0.00	182.06	2
sq42	100	0	0	0	0	2.30	2.80	3.89	3.75	6.67	6.65	12878.88	12269.50	0.00	0.00	289.70	5
sq43	100	23	0	0	0	0.60	1.20	0.74	0.70	3.02	2.90	6901.26	6801.00	0.03	0.00	166.81	1
sq44	100	6	0	0	0	0.10	0.90	1.30	1.20	2.64	2.55	6531.29	6417.50	0.09	0.00	156.84	1
sq45	100	17	0	0	0	0.00	0.20	0.62	0.50	1.44	1.45	6637.74	5920.00	0.30	0.00	147.20	0
sq46	100	2	0	0	0	1.00	1.20	1.97	1.95	3.13	3.05	7283.80	6953.00	0.02	0.01	181.38	1
sq47	100	63	0	0	0	0.00	0.70	0.12	0.00	2.08	2.30	7732.64	6780.00	0.22	0.00	178.27	0
sq48	100	1	0	0	0	2.00	4.30	5.07	5.20	8.65	8.70	9143.70	8535.50	0.00	0.00	249.03	4
sq49	100	53	9	0	0	0.00	0.20	0.23	0.00	0.98	0.75	6428.33	5834.00	0.48	0.01	151.69	0

sq50	100	26	0	0	0	0.00	0.20	0.51	0.50	2.23	2.40	6363.72	5870.50	0.12	0.00	144.42	0
sq51	100	0	0	0	0	4.50	8.30	10.03	9.75	14.70	14.75	7018.94	6673.00	0.00	87.27	233.70	12
sq52	100	1	0	0	0	1.70	3.10	2.65	2.50	7.41	7.50	8101.18	7775.50	0.00	0.00	241.83	4
sq53	100	79	4	0	0	0.00	0.10	0.10	0.00	1.09	1.00	7633.86	7340.50	0.55	0.00	187.27	1
sq54	100	0	0	0	0	2.30	3.60	4.73	4.80	7.10	7.10	9548.92	9327.00	0.00	0.05	246.69	6
sq55	100	5	0	0	0	0.10	1.50	1.29	1.10	3.00	2.80	10526.75	10247.00	0.03	0.00	256.75	1
sq56	100	0	0	0	0	2.10	2.60	3.80	3.65	5.83	5.85	9434.16	9225.00	0.00	0.00	247.34	3
sq57	100	48	2	0	0	0.20	0.80	0.29	0.15	2.11	2.20	6805.85	6176.50	0.19	0.00	165.91	1
sq58	100	0	0	0	0	0.10	0.80	2.51	2.20	4.04	3.90	9235.33	9054.50	0.02	0.00	236.55	1
sq59	100	2	0	0	0	0.00	1.80	3.16	3.20	5.17	5.00	10804.93	10002.00	0.06	0.02	262.15	5
sq60	100	0	0	0	0	3.40	4.10	6.42	6.20	8.36	8.05	9291.52	8451.00	0.00	0.00	237.74	5
sq61	100	3	0	0	0	0.40	0.60	1.37	1.30	2.47	2.20	6120.63	5988.00	0.04	0.00	162.91	1
sq62	100	3	0	0	0	0.90	1.50	2.41	2.50	5.50	5.35	9477.27	8898.00	0.02	0.00	240.81	4
sq63	100	85	3	2	0	0.00	0.10	0.06	0.00	1.05	1.00	6974.64	6704.00	0.27	0.00	160.11	1
sq64	100	0	0	0	0	0.30	0.80	4.82	4.90	6.72	6.60	7698.47	7467.00	0.02	0.00	214.51	4
sq65	100	2	0	0	0	1.10	2.00	2.56	2.55	5.09	5.10	8350.45	7688.00	0.01	0.00	231.70	3
sq66	100	11	0	0	0	0.00	1.20	1.43	1.20	3.40	3.10	7893.92	7655.50	0.09	0.00	213.50	2
sq67	100	0	0	0	0	1.70	4.30	4.91	4.80	9.17	9.10	7524.53	7470.00	0.00	0.01	225.66	5
sq68	100	3	0	0	0	0.50	2.40	2.93	2.70	6.89	6.85	8046.23	7704.50	0.00	0.01	234.49	4
sq69	100	0	0	0	0	0.60	6.90	8.46	8.50	12.67	12.25	6120.45	5843.50	0.00	6.00	197.66	10
sq70	100	86	26	1	0	0.00	0.40	0.04	0.00	1.27	1.00	5584.74	5475.00	0.29	0.00	134.45	0
sq71	100	78	11	0	0	0.00	1.10	0.08	0.00	3.07	2.90	6819.99	6293.00	0.07	0.00	168.24	0
sq72	100	25	2	0	0	0.10	0.10	0.74	0.60	1.68	1.55	6050.92	5871.00	0.05	0.00	152.68	1
sq73	100	7	0	0	0	0.00	1.70	1.62	1.50	3.75	3.55	11254.62	10838.00	0.16	0.00	281.52	3
sq74	100	4	0	0	0	0.50	1.00	1.73	1.65	4.21	4.20	11157.47	10327.50	0.04	0.00	276.41	3
sq75	100	20	1	0	0	0.10	0.30	0.86	0.80	2.43	2.30	8990.84	8706.00	0.08	0.00	228.23	2
sq76	100	0	0	0	0	0.80	3.60	4.46	4.60	7.12	7.05	7761.07	7004.50	0.00	0.00	219.19	3
sq77	100	1	0	0	0	0.00	0.90	2.25	2.20	3.74	3.80	7804.50	7213.00	0.04	0.00	200.62	1
sq78	100	1	0	0	0	1.40	4.50	4.13	4.00	9.38	9.10	9087.38	8932.50	0.00	5.59	252.72	10
sq79	100	5	0	0	0	2.10	2.60	2.34	2.20	6.37	6.30	8310.17	7803.50	0.00	0.01	248.67	5
sq80	100	99	1	0	0	0.00	0.40	0.00	0.00	0.86	0.80	6328.88	5673.50	0.29	0.00	151.96	0
sq81	100	0	0	0	0	3.70	5.90	4.85	4.50	10.24	10.50	8385.73	7724.00	0.00	0.01	239.02	4
sq82	100	86	12	5	2	0.00	0.00	0.06	0.00	0.84	0.70	6291.30	6136.00	0.17	0.00	156.94	1
sq83	100	26	2	0	0	0.00	0.30	0.62	0.45	1.56	1.50	7059.81	6874.50	0.09	0.00	158.64	1
sq84	100	11	3	2	0	0.00	0.20	0.81	0.70	1.83	1.75	5831.79	5639.00	0.19	0.00	153.44	2
sq85	100	0	0	0	0	0.10	2.50	3.09	3.00	5.44	5.15	6862.03	6648.50	0.03	0.00	193.01	2
sq86	100	6	0	0	0	0.20	0.50	1.62	1.50	2.78	2.80	8441.08	8123.50	0.08	0.00	211.64	2
sq87	100	33	1	0	0	0.10	1.00	0.37	0.20	2.98	2.90	8004.00	7104.00	0.12	0.00	190.71	1
sq88	100	19	2	0	0	1.00	1.60	0.83	0.70	3.04	2.90	6663.26	6216.50	0.07	0.00	154.29	1
sq89	100	63	5	1	0	0.00	0.10	0.19	0.00	1.09	1.00	5485.78	5196.00	0.17	0.01	138.94	2
sq90	100	73	6	1	0	0.20	0.60	0.09	0.00	2.26	2.20	6627.58	6184.50	0.12	0.00	148.26	1
sq91	100	49	6	1	0	0.00	0.20	0.38	0.10	1.58	1.40	9550.33	9248.50	0.06	0.00	245.08	4
sq92	100	0	0	0	0	3.30	7.70	7.12	7.05	12.43	12.45	7151.99	6737.50	0.00	0.03	133.44	6
sq93	100	2	0	0	0	1.10	1.40	1.99	1.90	4.91	4.80	9986.64	9377.50	0.02	0.00	163.46	2
sq94	100	35	4	0	0	0.00	0.20	0.41	0.20	1.32	1.30	6049.44	5813.00	0.19	0.01	91.66	0
sq95	100	0	0	0	0	5.80	7.60	9.06	9.05	13.01	13.20	6065.87	5951.00	0.00	1.44	112.62	9
sq96	100	16	0	0	0	0.00	0.40	0.68	0.60	2.12	2.05	7191.67	6812.00	0.05	0.00	101.05	2

sq97	100	67	1	0	0	0.00	0.20	0.22	0.00	2.30	2.20	5925.38	5806.00	0.16	0.00	83.26	2
sq98	100	84	0	0	0	0.00	0.80	0.04	0.00	2.11	2.10	6817.99	6247.00	0.18	0.00	95.28	2
sq99	100	25	13	3	3	0.00	0.00	0.42	0.40	0.70	0.70	6207.71	6054.00	0.12	0.00	84.98	0
sq100	100	1	0	0	0	0.00	2.60	2.79	2.80	5.84	5.70	11922.50	11469.50	0.01	0.00	206.90	3
μ		27	2	0	0	0.75	1.69							0.10			
$\tilde{x}$		10	0	0	0	0.10	0.90							0.06			

Supplementary Table 5: Detailed results of two-target pseudknot design inputs (LE80 dataset).

RNA	l	n1	n2	d1	d2	μ d1	$\tilde{x}$ d1	μ d2	$\tilde{x}$ d2	μ nom	$\tilde{x}$ nom	$\sum$ prob	μ constructing	μ sampling	max dim
PKB00002 PKB00004 0	50	11	5	0.00	0.00	0.57	0.15	0.71	0.35	635.77	638.50	0.24	0.00	3.41	0
PKB00005 PKB00015 0	41	0	0	0.60	0.60	1.42	1.40	1.63	1.70	568.57	534.50	0.10	0.00	3.03	0
PKB00008 PKB00031 0	40	0	0	0.20	0.40	1.34	1.10	1.67	1.40	656.43	579.50	0.19	0.01	3.46	0
PKB00010 PKB00066 0	40	12	5	0.00	0.00	0.52	0.20	0.70	0.45	593.07	573.50	0.38	0.00	3.11	0
PKB00012 PKB00268 0	40	8	4	0.00	0.00	0.85	0.65	0.95	0.70	453.80	399.00	0.05	0.00	2.39	0
PKB00030 PKB00045 0	41	0	0	0.70	0.90	1.64	1.40	1.85	1.60	588.83	529.00	0.19	0.01	3.08	0
PKB00047 PKB00069 0	61	0	0	3.00	3.30	5.61	5.45	5.80	5.70	487.47	449.00	0.00	0.00	2.61	0
PKB00048 PKB00265 0	61	0	0	1.10	1.20	3.40	3.10	6670.27	3.65	517.87	452.00	0.01	0.00	2.75	0
PKB00050 PKB00128 0	59	10	4	0.00	0.00	0.65	0.40	0.93	0.75	518.03	481.50	0.15	0.00	2.71	0
PKB00052 PKB00107 0	52	4	1	0.00	0.00	6667.73	0.85	6667.99	1.15	437.93	385.50	0.25	0.00	2.33	0
PKB00057 PKB00072 0	67	0	0	1.30	2.10	5.41	5.30	5.69	5.50	495.33	436.00	0.00	0.00	2.59	0
PKB00068 PKB00129 0	68	0	0	2.60	3.30	5.34	5.10	5.65	5.45	681.17	640.50	0.00	0.00	3.41	0
PKB00070 PKB00244 0	55	2	0	0.00	0.10	1.91	1.75	2.32	1.90	481.70	394.50	0.17	0.00	2.52	0
PKB00078 PKB00106 0	62	4	0	0.00	0.10	1.17	1.00	2.25	1.90	525.20	466.50	0.24	0.00	2.77	0
PKB00080 PKB00132 0	49	10	4	0.00	0.00	0.50	0.40	0.70	0.60	407.60	407.50	0.07	0.00	2.11	0
PKB00088 PKB00127 0	62	10	1	0.00	0.00	0.74	0.55	1.54	1.15	676.53	658.00	0.25	0.00	3.58	0
PKB00098 PKB00232 0	62	1	0	0.00	0.40	2.53	2.65	2.80	2.80	609.90	580.00	0.04	0.00	3.64	0
PKB00131 PKB00205 0	48	0	0	1.70	2.20	3.02	3.00	4.16	3.90	570.73	567.50	0.01	0.00	3.35	0
PKB00139 PKB00141 0	70	0	0	1.50	1.60	3.17	3.00	3.27	3.20	745.73	662.00	0.01	0.00	3.89	0
PKB00142 PKB00231 0	71	1	0	0.00	0.80	2.86	2.55	3.33	2.90	498.97	468.50	0.05	0.00	2.75	0
PKB00143 PKB00233 0	71	0	0	1.40	1.50	13337.10	3.70	13337.27	3.75	603.03	577.00	0.01	0.00	3.22	2
PKB00148 PKB00218 0	72	0	0	3.30	3.60	5.52	4.95	5.77	5.10	642.50	571.00	0.00	0.00	3.35	0
PKB00175 PKB00259 0	57	0	0	0.30	0.50	1.69	1.65	1.98	1.90	648.77	643.00	0.08	0.00	3.95	0
PKB00179 PKB00280 0	68	0	0	0.60	0.60	2.61	2.70	2.90	2.90	565.13	587.50	0.01	0.00	3.05	0
PKB00180 PKB00212 0	64	0	0	0.30	0.40	6670.49	3.05	20003.69	3.65	448.53	402.50	0.13	0.00	2.62	0
PKB00190 PKB00266 0	47	21	7	0.00	0.00	0.18	0.00	0.34	0.20	534.43	530.00	0.29	0.00	3.19	0
PKB00207 PKB00213 0	45	7	1	0.00	0.00	13334.10	0.60	13334.26	0.85	364.37	339.50	0.26	0.00	2.17	0
PKB00211 PKB00239 0	80	0	0	0.80	1.10	4.15	3.75	4.62	4.50	486.80	464.00	0.02	0.00	2.27	0
PKB00222 PKB00305 0	80	0	0	0.50	0.90	6670.15	3.35	6670.54	3.85	595.60	592.00	0.02	0.00	2.61	0
PKB00224 PKB00281 0	43	9	3	0.00	0.00	0.70	0.55	1.01	0.75	513.63	485.00	0.19	0.00	3.05	0
PKB00230 PKB00273 0	48	0	0	2.00	2.50	4.11	4.10	6671.29	4.65	374.43	353.00	0.00	0.00	2.19	0
PKB00248 PKB00257 0	66	0	0	4.40	6.70	6675.06	8.55	33343.25	10.90	214.17	221.00	0.00	0.00	1.20	0
PKB00263 PKB00270 0	62	6	1	0.00	0.00	13334.31	0.95	13334.51	1.15	620.23	629.50	0.16	0.00	3.11	0

PKB00269	PKB00272	0	66	0	0	1.50	2.30	6670.63	3.80	20004.32	4.40	444.57	411.00	0.00	0.00	1.25	0
μ				3	1	0.82	1.09							0.11			
$\tilde{x}$				0	0	0.30	0.55							0.06			

Supplementary Table 6: Detailed results of two-target pseudknot design inputs (PK60 dataset).

RNA	l	n1	n2	d1	d2	μ d1	$\tilde{x}$ d1	μ d2	$\tilde{x}$ d2	μ nom	$\tilde{x}$ nom	$\sum$ prob	μ constructing	μ sampling	max dim
no1	60	0	0	0.20	0.20	1.64	1.35	1.82	1.60	620.13	659.00	0.11	0.00	3.26	0
no2	60	2	0	0.20	0.30	1.48	1.55	1.87	1.70	1015.93	1008.50	0.06	0.00	6.12	0
no3	60	17	5	0.00	0.00	0.39	0.00	0.65	0.20	806.70	707.00	0.12	0.00	4.85	2
no4	60	11	5	0.00	0.00	0.58	0.20	0.70	0.30	810.63	769.00	0.14	0.00	4.44	0
no5	60	3	1	0.00	0.00	1.20	1.30	1.41	1.40	696.63	686.50	0.08	0.00	3.99	0
no6	60	2	0	0.00	0.30	1.16	0.95	1.40	1.30	787.03	736.00	0.15	0.00	4.29	0
no7	60	29	18	0.00	0.00	0.04	0.00	0.11	0.00	929.33	897.50	0.45	0.00	4.70	0
no8	60	0	0	0.30	0.30	2.50	2.30	2.72	2.60	647.67	651.00	0.06	0.00	3.32	0
no9	60	27	11	0.00	0.00	0.03	0.00	0.16	0.10	908.17	887.00	0.27	0.00	2.53	0
no10	60	21	9	0.00	0.00	0.15	0.00	0.31	0.10	944.97	917.00	0.39	0.01	5.07	0
no11	60	27	15	0.00	0.00	0.05	0.00	0.17	0.05	862.27	806.00	0.41	0.00	4.65	0
no12	60	17	3	0.00	0.00	0.38	0.00	0.61	0.55	779.47	779.00	0.33	0.00	4.24	0
no13	60	8	1	0.00	0.00	0.45	0.25	0.56	0.40	784.07	791.00	0.17	0.00	4.24	0
no14	60	28	14	0.00	0.00	0.03	0.00	0.11	0.10	852.70	798.50	0.28	0.00	4.59	0
no15	60	3	1	0.00	0.00	1.31	1.25	1.57	1.50	615.13	595.00	0.18	0.00	3.31	0
no16	60	7	2	0.00	0.00	0.60	0.45	0.77	0.55	729.23	736.00	0.17	0.00	3.95	0
no17	60	0	0	0.20	0.50	1.98	1.90	2.17	2.15	657.07	619.00	0.06	0.00	3.50	0
no18	60	25	7	0.00	0.00	0.15	0.00	0.32	0.20	719.77	717.50	0.46	0.00	3.85	0
no19	60	14	3	0.00	0.00	0.62	0.10	0.89	0.55	784.93	683.50	0.27	0.00	4.20	0
no20	60	4	0	0.00	0.10	1.14	0.85	1.44	1.30	652.77	640.50	0.19	0.00	3.49	0
no21	60	3	1	0.00	0.00	1.71	1.70	1.94	1.85	592.00	576.50	0.09	0.00	3.11	0
no22	60	23	7	0.00	0.00	0.10	0.00	0.27	0.20	880.83	885.50	0.31	0.00	4.65	0
no23	60	30	15	0.00	0.00	0.00	0.00	0.11	0.05	881.77	860.50	0.75	0.00	4.65	0
no24	60	3	0	0.00	0.20	1.21	1.20	1.43	1.35	532.30	486.50	0.37	0.00	2.81	0
no25	60	20	15	0.00	0.00	0.13	0.00	0.18	0.05	841.13	822.00	0.27	0.00	5.12	0
no26	60	28	11	0.00	0.00	0.04	0.00	0.14	0.10	845.93	900.50	0.44	0.00	5.18	0
no27	60	7	1	0.00	0.00	1.44	1.30	1.92	1.45	633.67	625.00	0.16	0.00	3.90	0
no28	60	27	4	0.00	0.00	0.02	0.00	0.26	0.15	902.00	877.50	0.45	0.00	5.45	0
no29	60	17	5	0.00	0.00	0.36	0.00	0.50	0.30	639.13	604.50	0.06	0.00	3.95	0
no30	60	22	6	0.00	0.00	0.15	0.00	0.30	0.20	672.63	652.00	0.14	0.00	4.07	0
no31	60	1	0	0.00	0.20	1.84	1.25	2.06	1.55	696.03	704.00	0.07	0.00	4.25	0
no32	60	23	3	0.00	0.00	0.14	0.00	0.30	0.20	905.87	876.00	0.32	0.00	5.45	0
no33	60	12	4	0.00	0.00	0.47	0.40	0.63	0.55	652.57	589.00	0.41	0.01	4.01	0
no34	60	9	4	0.00	0.00	0.75	0.50	0.86	0.55	528.40	475.50	0.14	0.00	3.27	0
no35	60	21	8	0.00	0.00	0.21	0.00	0.39	0.40	844.40	802.00	0.46	0.00	5.18	0
no36	60	9	1	0.00	0.00	0.59	0.40	0.83	0.55	805.07	812.00	0.33	0.00	4.69	0

RNA	l	n1	n2	d1	d2	μ d1	$\tilde{x}$ d1	μ d2	$\tilde{x}$ d2	μ nom	$\tilde{x}$ nom	$\sum$ prob	μ constructing	μ sampling	max dim
no37	60	0	0	0.60	1.00	1.88	1.80	2.16	2.00	937.03	1000.50	0.03	0.00	5.69	0
no38	60	1	1	0.00	0.00	1.55	1.25	1.79	1.55	647.67	640.00	0.24	0.00	3.96	0
no39	60	30	18	0.00	0.00	0.00	0.00	0.06	0.00	865.63	862.00	0.48	0.00	5.33	0
no40	60	24	15	0.00	0.00	0.13	0.00	0.27	0.05	1026.00	1072.00	0.27	0.00	6.07	0
no41	60	22	9	0.00	0.00	0.13	0.00	0.34	0.10	810.83	743.00	0.22	0.00	4.89	0
no42	60	26	7	0.00	0.00	0.09	0.00	0.22	0.10	885.23	835.00	0.45	0.00	5.27	0
no43	60	2	0	0.00	0.80	2.10	1.90	2.52	2.40	539.30	485.00	0.05	0.00	3.24	0
no44	60	29	9	0.00	0.00	0.01	0.00	0.15	0.10	1038.80	1084.00	0.46	0.00	6.08	0
no45	60	13	4	0.00	0.00	0.76	0.35	1.01	0.60	828.50	831.00	0.12	0.00	4.91	0
no46	60	4	1	0.00	0.00	1.51	1.10	1.78	1.60	437.30	422.50	0.05	0.00	2.67	0
no47	60	2	0	0.00	0.10	1.90	1.80	2.14	2.05	645.33	591.00	0.03	0.00	3.87	0
no48	60	0	0	0.10	0.10	1.52	1.30	1.72	1.60	695.47	681.00	0.12	0.00	4.11	2
no49	60	18	7	0.00	0.00	0.17	0.00	0.29	0.15	878.80	829.00	0.32	0.00	4.90	0
no50	60	18	7	0.00	0.00	0.35	0.00	0.53	0.25	666.67	657.50	0.10	0.00	3.78	0
μ		14	5	0.03	0.08							0.24			
$\tilde{x}$		14	4	0.00	0.00							0.21			

Supplementary Table 7: Detailed results of two-target pseudknot design inputs (PK80 dataset).

RNA	l	n1	n2	d1	d2	μ d1	$\tilde{x}$ d1	μ d2	$\tilde{x}$ d2	μ nom	$\tilde{x}$ nom	$\sum$ prob	μ constructing	μ sampling	max dim
no1	80	3	2	0.00	0.00	1.13	1.00	1.31	1.30	871.63	915.00	0.09	0.00	4.80	0
no2	80	23	15	0.00	0.00	0.15	0.00	0.20	0.05	846.37	799.00	0.17	0.00	4.74	0
no3	80	28	4	0.00	0.00	0.05	0.00	0.19	0.10	1178.70	1119.50	0.22	0.00	6.33	0
no4	80	22	8	0.00	0.00	0.18	0.00	0.34	0.10	1007.97	940.00	0.17	0.00	5.56	0
no5	80	12	7	0.00	0.00	0.64	0.25	0.74	0.40	1079.90	1108.50	0.27	0.00	5.99	0
no6	80	25	18	0.00	0.00	0.20	0.00	0.25	0.00	971.53	933.50	0.10	0.01	5.34	0
no7	80	5	1	0.00	0.00	0.96	0.80	1.12	0.95	833.23	816.00	0.12	0.00	4.70	0
no8	80	1	0	0.00	0.20	1.80	1.45	2.00	1.70	847.50	803.00	0.14	0.00	4.80	0
no9	80	20	14	0.00	0.00	0.29	0.00	0.34	0.10	931.57	895.50	0.19	0.00	2.43	0
no10	80	20	8	0.00	0.00	0.27	0.00	0.38	0.15	925.50	929.00	0.26	0.00	5.17	0
no11	80	29	10	0.00	0.00	0.01	0.00	0.15	0.10	1083.83	1006.00	0.21	0.00	6.01	0
no12	80	30	20	0.00	0.00	0.00	0.00	0.06	0.00	1006.93	959.00	0.42	0.00	5.59	0
no13	80	30	16	0.00	0.00	0.00	0.00	0.08	0.00	1114.80	1123.00	0.41	0.00	6.15	0
no14	80	10	3	0.00	0.00	0.59	0.55	0.77	0.65	1087.57	1057.00	0.22	0.00	6.01	0
no15	80	25	5	0.00	0.00	0.08	0.00	0.18	0.10	1098.63	1064.50	0.14	0.00	6.02	0
no16	80	27	9	0.00	0.00	0.02	0.00	0.18	0.10	971.37	941.50	0.31	0.00	5.38	0
no17	80	8	4	0.00	0.00	0.62	0.55	0.73	0.65	1064.17	1107.50	0.12	0.00	5.86	0
no18	80	18	8	0.00	0.00	0.29	0.00	0.39	0.20	842.50	760.50	0.10	0.00	4.63	0
no19	80	29	13	0.00	0.00	0.00	0.00	0.08	0.10	1164.00	1130.50	0.24	0.00	6.42	0
no20	80	17	7	0.00	0.00	0.31	0.00	0.40	0.20	1178.23	1212.50	0.24	0.00	6.38	0
no21	80	1	0	1.10	1.30	6671.48	4.45	6671.74	4.60	707.30	652.00	0.01	0.00	3.84	0
no22	80	7	1	0.00	0.00	0.85	0.60	1.09	0.85	1144.57	1137.50	0.25	0.00	6.24	0
no23	80	29	13	0.00	0.00	0.04	0.00	0.18	0.10	1181.20	1121.00	0.53	0.00	6.40	0

no24	80	7	2	0.00	0.00	0.98	0.60	1.16	0.75	835.43	815.00	0.29	0.00	4.51	0
no25	80	7	2	0.00	0.00	1.04	1.10	1.43	1.40	776.83	709.50	0.31	0.00	4.57	0
no26	80	0	0	3.30	3.40	5.81	5.55	6.51	5.95	531.07	508.50	0.00	0.00	3.27	0
no27	80	1	0	0.00	0.20	2.92	2.65	3.37	3.30	979.33	905.50	0.12	0.00	5.75	0
no28	80	24	12	0.00	0.00	0.15	0.00	0.34	0.10	1146.77	1176.50	0.10	0.00	6.71	0
no29	80	21	7	0.00	0.00	0.18	0.00	0.31	0.15	989.97	974.50	0.12	0.00	5.50	0
no30	80	14	3	0.00	0.00	0.30	0.10	0.58	0.35	965.67	980.00	0.22	0.01	5.35	0
μ		16	7	0.15	0.17							0.20			
$\tilde{x}$		19	7	0.00	0.00							0.20			

References

[1] M. Andronescu, A. P. Fejes, F. Hutter, H. H. Hoos, and A. Condon. A new algorithm for RNA secondary structure design. *J Mol Biol*, 336(3):607–624, Feb 2004.

[2] A. Avihoo, A. Churkin, and D. Barash. RNAexinv: An extended inverse rna folding from shape and physical attributes to sequences. *BMC Bioinformatics*, 12(1):319, 2011.

[3] A. Busch and R. Backofen. INFO-RNA – a fast approach to inverse RNA folding. *Bioinformatics*, 22(15), Aug. 2006.

[4] K. Darty, A. Denise, and Y. Ponty. VARNA: Interactive drawing and editing of the RNA secondary structure. *Bioinformatics*, 25(15):1974–1975, Aug 2009.

[5] R. M. Dirks, M. Lin, E. Winfree, and N. A. Pierce. Paradigms for computational nucleic acid design. *Nucleic Acids Res*, 32(4):1392–1403, 2004.

[6] A. Esmaili-Taheri, M. Ganjtabesh, and M. Mohammad-Noori. Evolutionary solution for the RNA design problem. *Bioinformatics*, 30(9):1250–1258, Jan 2014.

[7] A. Espah-Borujeni, D. M. Mishler, J. Wang, W. Huso, and H. M. Salis. Automated physics-based design of synthetic riboswitches from diverse RNA aptamers. *Nucleic Acids Research*, page gkv1289, Nov 2015.

[8] C. Flamm, I. L. Hofacker, S. Maurer-Stroh, P. F. Stadler, and M. Zehl. Design of multistable RNA molecules. *RNA*, 7(2):254–265, Feb. 2001.

[9] J. A. Garcia-Martin, P. Clote, and I. Dotu. RNAiFOLD: a constraint programming algorithm for rna inverse folding and molecular design. *J Bioinform Comput Biol*, 11(2):1350001, Apr 2013.

[10] J. A. Garcia-Martin, P. Clote, and I. Dotu. RNAiFold: a web server for rna inverse folding and molecular design. *Nucleic Acids Res*, 41(Web Server issue):W465–W470, Jul 2013.

[11] J. A. Garcia-Martin, I. Dotu, and P. Clote. RNAiFold 2.0: a web server and software to design custom and rfam-based RNA molecules. *Nucleic Acids Research*, 43(W1):W513–W521, may 2015.

[12] I. L. Hofacker, W. Fontana, P. F. Stadler, L. S. Bonhoeffer, M. Tacker, and P. Schuster. Fast folding and comparison of RNA secondary structures. *Monatshefte für Chemie / Chemical Monthly*, 125(2):167–188, Feb. 1994.

[13] C. Höner zu Siederdissen, S. Hammer, I. Abfalter, I. L. Hofacker, C. Flamm, and P. F. Stadler. Computational design of RNAs with complex energy landscapes. *Biopolymers*, 99(12):1124–1136, 2013.

[14] R. Kleinkauf, T. Houwaart, R. Backofen, and M. Mann. antaRNA – Multi-objective inverse folding of pseudoknot RNA using ant-colony optimization. *BMC Bioinformatics*, 16, 2015.

[15] R. Kleinkauf, M. Mann, and R. Backofen. antaRNA: ant colony-based RNA sequence design. *Bioinformatics*, 31(19):31143121, May 2015.

[16] J. Lee, W. Kladwang, M. Lee, D. Cantu, M. Azizyan, H. Kim, A. Limpaecher, S. Yoon, A. Treuille, and R. Das. RNA design rules from a massive open laboratory. *Proceedings of the National Academy of Sciences*, pages 2122–2127, Jan 2014.

[17] A. Levin, M. Lis, Y. Ponty, C. W. O'Donnell, S. Devadas, B. Berger, and J. Waldispühl. A global sampling approach to designing and reengineering RNA secondary structures. *Nucleic Acids Res*, 40(20):10041–10052, Nov 2012.

[18] R. B. Lyngso, J. W. Anderson, E. Sizikova, A. Badugu, T. Hyland, and J. Hein. Frnakenstein: multiple target inverse RNA folding. *BMC Bioinformatics*, 13(1):260, 2012.

[19] M. C. Matthies, S. Bienert, and A. E. Torda. Dynamics in sequence space for RNA secondary structure design. *Journal of Chemical Theory and Computation*, 8(10):3663–3670, Oct 2012.

[20] V. Reinharz, Y. Ponty, and J. Waldispühl. A weighted sampling algorithm for the design of RNA sequences with targeted secondary structure and nucleotide distribution. *Bioinformatics*, 29(13), Jul 2013.

[21] G. Rodrigo and A. Jaramillo. RiboMaker: computational design of conformation-based riboregulation. *Bioinformatics*, 30(17):2508–2510, may 2014.

[22] W. Shu, M. Liu, H. Chen, X. Bo, and S. Wang. ARDesigner: a web-based system for allosteric RNA design. *J Biotechnol*, 150(4):466–473, Dec 2010.

[23] A. Taneda. MODENA: a multi-objective RNA inverse folding. *Adv Appl Bioinform Chem*, 4:1–12, 2011.

[24] A. Taneda. Multi-objective optimization for RNA design with multiple target secondary structures. *BMC Bioinformatics*, 16(1):280, Sept. 2015.

[25] B. R. Wolfe and N. A. Pierce. Sequence Design for a Test Tube of Interacting Nucleic Acid Strands. *ACS Synthetic Biology*, 4(10):1086–1100, Oct. 2015.
